# Transcriptional Analysis of a Unique Set of Genes Involved in *Schistosoma mansoni* Female Reproductive Biology

**DOI:** 10.1371/journal.pntd.0001907

**Published:** 2012-11-15

**Authors:** Alexis A. Cogswell, Valerie P. Kommer, David L. Williams

**Affiliations:** Department of Immunology/Microbiology, Rush University Medical Center, Chicago, Illinois, United States of America; University of Queensland, Australia

## Abstract

Schistosomiasis affects more than 200 million people globally. The pathology of schistosome infections is due to chronic tissue inflammation and damage from immune generated granulomas surrounding parasite eggs trapped in host tissues. *Schistosoma* species are unique among trematode parasites because they are dioecious; females require paring with male parasites in order to attain reproductive maturity and produce viable eggs. *Ex vivo* cultured females lose the ability to produce viable eggs due to an involution of the vitellarium and loss of mature oocytes. In order to better understand schistosome reproductive biology we used data generated by serial analysis of gene expression (SAGE) to identify uncharacterized genes which have different transcript abundance in mature females, those that have been paired with males, and immature females obtained from unisexual infections. To characterize these genes we used bioinformatics, transcript localization, and transcriptional analysis during the regression of *in vitro* cultured females. Genes transcribed exclusively in mature females localize primarily in the vitellocytes and/or the ovary. Genes transcribed exclusively in females from single sex infections localize to vitellocytes and subtegumental cells. As female reproductive tissues regress, eggshell precursor proteins and genes involved in eggshell synthesis largely have decreased transcript abundance. However, some genes with elevated transcript abundance in mature adults have increased gene expression following regression indicating that the genes in this study function both in eggshell biology as well as vitellogenesis and maintenance of female reproductive tissues. In addition, we found that genes enriched in females from single sex infections have increased expression during regression in *ex vivo* females. By using these transcriptional analyses we can direct research to examine the areas of female biology that are both relevant to understanding the overall process of female development and worm pairing while determining novel therapeutic approaches directed at the maturation of female schistosomes.

## Author Summary

Schistosomiasis is a chronic, debilitating disease that affects over 200 million people globally. The pathology associated with schistosomiasis is caused by host immune responses to parasite eggs. Therefore, it is imperative to identify pathways responsible for controlling worm reproductive biology. Schistosome females must be in constant contact with male parasites in order to achieve reproductive maturity. The process of pairing and reproductive maturation in female worms is poorly understood, in part, because it does not occur outside of the host. In addition, when female schistosomes are removed from their mammalian host they regress to an immature state. In this study our goal was to characterize a unique set of genes in *Schistosoma mansoni* whose transcript abundance differs in mature and immature female worms. We found that the genes with higher transcript abundance in sexually mature female worms were expressed in female reproductive tissues, while those transcripts enriched in sexually immature worms were present in sub-surface somatic cells. Transcript abundance of the selected genes changed dramatically when females were removed from their host. These findings inform new approaches to study female worm biology and will provide insights into the processes of worm pairing and reproductive maturation.

## Introduction

Schistosomiasis, caused by infections with trematode parasites from the genus *Schistosoma*, affects more than 200 million people worldwide [1]. Cercariae released by intermediate host snails penetrate the skin of the mammalian definitive host where they develop into schistosomula. Schistosomula migrate first to the lungs and then to the liver sinusoids. In the liver juvenile parasites begin blood feeding and pair prior to migrating to the mesenteric or rectal veins (*S. mansoni* and *S. japonicum*) or to the perivesical venous plexus of the bladder (*S. hematobium*) where they mature into adult parasites. Paired worms produce eggs that pass out of the host in the urine or feces. These eggs can also become trapped in immune-generated granulomas in host tissues, primarily the liver, bladder wall and intestinal epithelium, causing a strong Th2 immune response resulting in the pathology associated with schistosomiasis. Since eggs are central to the pathology of schistosomiasis and the transmission of the parasite to the intermediate host it is essential to improve our understanding of worm reproductive biology.

Schistosomes are unique among trematodes because they are dioecious, with worm sex being determined chromosomally [2]. Furthermore, females must pair with a male in order undergo complete sexual development and the production of infectious eggs. After pairing with a male, the female reproductive structures, mainly the ovary and the vitelline glands, undergo terminal differentiation. The vitellarium comprises two thirds of the mature female body and participates in a diverse array of functions. Vitellocytes undergo four distinct developmental phases. Stage 1 cells are the most immature and localize to the distal regions of the vitellarium farthest away from the vitelline duct. They possess few lipid droplets and ribosomes, both requirements for the synthesis of eggshell precursor proteins. The progenitor-like state of Stage 1 vitellocytes suggests that they are a stem cell population that undergoes mitosis and differentiation when females pair with males [3], [4]. Stage 2 and 3 vitellocytes represent intermediate stages of differentiation in which they are directed to a terminal cell fate, but are still synthesizing the necessary components required for mature vitellocyte functions. Stage 4 vitellocytes are terminally differentiated and are the largest cells in the vitellarium. They possess large lipid droplets and a large number of ribosomes. These cells contain vesicles with proteins that are required for eggshell synthesis, including shell precursor proteins and tyrosinase, an enzyme that cross-links the precursor proteins to form the eggshell, and are localized proximal to the vitelline duct allowing for their release into the duct which they traverse to reach the ootype where they merge with an oocyte and egg biogenesis begins.

The ovary also undergoes dramatic changes as maturation occurs. Unpaired females posses a highly coiled immature ovary entirely composed of oogonia, immature female germ cells [3]. Upon pairing, the oogonia undergo mitosis and meiosis to produce mature oocytes. Mature oocytes are round in structure and the nucleus is small compared to the cytoplasm [4], [5]. In mature females, the oogonia are restricted to the anterior ovary whereas the oocytes reside in the posterior ovary where they are released into the oviduct. As the oocytes traverse the oviduct they merge with vitellocytes in the ovo-vitelline duct leading to the ootype, where the egg is formed [5], [6].

The ootype, surrounded by the Mehlis' gland, is where the vitellocytes, sperm, and oocyte form an egg. The process of eggshell formation is rapid due to the presence of pre-made precursor proteins, but the signals leading to their release and eggshell formation have not yet been characterized. Egg formation is aided by secretions from the Mehlis' gland [7]. In addition, the vitellocytes are responsible for the synthesis of two subshell layers; the Reynolds' layer, localized proximally to the eggshell, and the von Lichtenberg's envelope, a syncytial epithelium surrounding the developing oocyte [7]. These subshell layers develop along with the miracidium after the egg is excreted from the gonopore.

Single sex schistosome infections do not occur in a natural setting, but can be generated by infecting laboratory hosts with cercariae produced from mono-miracidial infections of snails. Because cercariae are produced by asexual reproduction in sporocysts, the gender of the infecting miracidium determines the gender of the cercariae produced. In this way, we are able to study differences between age-matched female worms that have developed in the absence of male worms (adult female worms from single-sex infections, AFSS) and sexually mature, egg-producing females that have paired with a male and undergone complete sexual development (adult female worms from mixed sex infections, AFMS). There are striking morphological and histological differences between AFSS and AFMS parasites [8], [9]. AFSS are smaller than AFMS, the size difference being largely reflective of the undifferentiated vitellarium. The ovary of AFSS worms is shrunken and contains only oogonia [10] indicating that female germ cells are present but in the absence of unknown, male-derived signals these germ cells are incapable of undergoing divisions to produce oocytes. The vitellarium of AFSS parasites is smaller than in AFMS parasites and contains only stage 1 vitellocytes [8], again reinforcing the notion that female germ cells are present in AFSS, but without proper signals, stage 1 vitellocytes are not able to mature. Biochemical differences include decreased production of eggshell precursor proteins (e.g., p14, p19, p48) and tyrosinase in AFSS compared to AFMS [11]–[14]. After mature worms are removed from their mammalian host and cultured *in vitro* they lose the ability to produce viable eggs and AFMS reproductive tissue regress to an immature stage similar to those found in AFSS [11], [15], 16. This process is termed “regression”.

Sequencing of the *S. mansoni* genome [17] has allowed research to shift from single gene-directed studies to global studies of various aspects of worm biology and pathogenesis. It is estimated that 40% of schistosome genes have no known function and have no homology to proteins in other genera [17]. Global transcription analysis during worm development using serial analysis of gene expression (SAGE) has been used to identify genes that have different transcript abundance during worm development and differentiation [18]. Here we investigate schistosome female reproductive biology and characterize genes differentially regulated in AFMS and AFSS worms through tissue localization and transcriptional analysis during seven days of regression. We find that many AFMS-enriched transcripts localize to the vitellarium and vitellocytes within the vitelline duct. We also find that while most genes are down-regulated in *ex vivo* females regardless of the presence of a male, removal of AFMS parasites from the host leads to an increase in transcript abundance for a select set of genes. Overall, these studies demonstrate that the involution of the vitellarium and the loss of reproductive maturity are governed by events occurring early during regression and provide evidence that female reproductive maturity is governed by signals provided both by the male parasite and the host microenvironment.

## Materials and Methods

### Ethics statement

This study was approved by the Institutional Animal Care and Use Committee at Rush University Medical Center (IACUC number 11-064; DHHS animal welfare assurance number A3120-01). Rush University Medical Center's Comparative Research Center (CRC) is operated in accordance with the Animal Welfare Act (Public Law (P.L.) 89–544) as amended by P.L.91–579 (1970); P.L.94–279 (1976); P.L. 99–198 (1985); and P.L 101–624 (1990), the Public Health Service's Policy on Humane Care and Use of Laboratory Animals (revised, 2002), the Guide for the Care and Use of Laboratory Animals (revised, 2011) and the U.S. Government Principles for the Utilization and Care of Vertebrate Animals Used in Testing, Research and Training. The CRC is registered with the Animal and Plant Health Inspection Service (APHIS) arm of the United States Department of Agriculture (USDA). The Institution has an Animal Welfare Assurance on file with the National Institutes of Health, Office of Laboratory Animal Welfare (OLAW), A-3120- 01. The facilities are accredited by the Association for Assessment and Accreditation of Laboratory Animal Care International (AAALAC International). The CRC is directed by the Senior Director of the CRC, a Doctor of Veterinary Medicine (D.V.M.) and a Diplomate of the American College of Laboratory Animal Medicine (ACLAM), who reports to the Associate Provost and Vice President for Research, who is also the Institutional Official for Animal Care and Use.

### Parasites and animals

The Puerto Rican/NMRI strain of *S. mansoni* and female Swiss-Webster mice were used in all experiments. Mice were euthanized using Nembutal in sterile water with heparin and adult schistosomes were recovered by hepatic portal perfusion [19]. Eggs were purified from the livers of infected mice and hatched in pond water to obtain miracidia for mono-miracidial infections of juvenile *Biomphalaria glabrata* snails (strain NMRI). Snails were shed in individual wells five weeks post infection and mice were infected with cercariae shed from a single snail via tail exposure as described [19]. Seven weeks post infection mice were euthanized and worms were obtained as described above.

### Identification of AFMS- and AFSS-enriched transcripts

Data on global transcription analysis during worm development generated by SAGE [18] was mined to identify transcripts with different steady-state abundance in AFMS and AFSS worms. The SAGE method captures 21-base pair tags that are used to uniquely identify the source gene from within the genome. Tags are isolated from the mRNA pool, concatenated, cloned, and sequenced. The population of tags defines patterns of expression of individual genes and quantification of all tags provides a relative measure of gene expression (i.e., mRNA abundance). A detailed description of SAGE can be found in [18]. SAGE tags were mapped to a predicted transcript using BLAST analysis of the available *S. mansoni* genomic (http://www.genedb.org/Homepage/Smansoni) and EST (http://compbio.dfci.harvard.edu) databases. To begin, we determined chromosomal location of the SAGE tag and then we identified any coding regions 5′ of the SAGE tag either predicted through GeneDB or confirmed through EST sequencing. To clone the sequences, SAGE tags (Table S1) were used as reverse primers in PCR along with the T3 primer present in pBluescript portion of the *S. mansoni* λ phage cDNA library to perform modified 5′ RACE PCR [20]. The product was cloned into pCRII vector and transformed into chemically competent Top10 *Escherichia coli* using the TOPO TA cloning kit (Invitrogen). In cases where the SAGE tag was associated with a predicted transcript we designed gene specific primers for PCR amplification. After sequence confirmation we designed additional primers in order to amplify a region of the gene to be used as a probe. Primers used for cloning, RT-PCR, and riboprobe synthesis are shown in Table S2. Protein domain identification and analysis was done using BLASTp, PSI-BLAST, and Interpro Scan.

### Life-cycle stage transcriptional analysis

cDNA was synthesized from total RNA obtained from separated adult male and female parasites from mixed-sex infections, male and female parasites obtained from single sex infections, all seven weeks post infection, and liver-stage parasites 23 days post infection. Worms were homogenized in Trizol (Ambion) using a sterile dounce and RNA was isolated using a chloroform extraction followed by ethanol precipitation. RNA was treated with DNAse (Promega) and cleaned using the Zymo DNA-free RNA kit (Zymo). Concentration and purity of RNA was verified using a Nanodrop spectrophotometer and cDNA was synthesized using the iScript cDNA synthesis kit (BioRad) using random hexamer and oligo(dT) primers. Gene-specific forward primers were designed from the sequences identified above and used with the SAGE tags to amplify transcripts from freshly made cDNA. For genes that were not identified by SAGE, gene specific primers designed from the predicted open reading frames identified in the *S. mansoni* GeneDB were used in RT-PCR reactions. β-tubulin was used as a control gene. Primers are shown in Table S2. Following cDNA synthesis, RT-PCR was performed using Flexi GoTaq DNA polymerase (Promega) under the following parameters: an initial denaturation step of 95°C 1 min followed by 25–35 cycles of 95°C 30 sec, 48–54°C 1 min, 72°C for 1 min, and a final extension of 72°C for 7 min. Both the cycle number and the annealing temperatures were dependent on the gene of interest (Table S2).

### Tissue localization of AFMS- and AFSS-enriched transcripts

Riboprobes were synthesized according to previously published methods [21]. Briefly, probes were synthesized from restriction enzyme digested DNA according to the orientation of the insert in pCRII using the Riboprobe synthesis kit (Promega) labeling with digoxigenin (Roche). Whole mount *in situ* hybridizations (WISH) were done as described [21]. Briefly, worms were fixed in 4% paraformaldehyde for 30 min and dehydrated in methanol. Following bleaching in 6% hydrogen peroxide in methanol to prevent tanning of the vitellaria, worms were permeabilized using Proteinase K (Ambion), incubated with pre-hybridization buffer (55% deionized formamide, 5× Saline Sodium Citrate (SSC), 1 mg/mL yeast RNA, 1% Tween 20) for 2 hours and then hybridized (pre-hybridization buffer with 10% dextran sulfate) with a riboprobe at 56°C overnight. Excess riboprobe was removed by washing in 2× and 0.2× SSC followed by blocking in K block (5% horse serum, 0.45% fish gelatin, 0.3% Triton-X 100, 0.05% Tween 20 in 1× PBS). Detection of bound riboprobe was done by incubation of worms in anti-digoxigenin alkaline phosphatase-conjugated antibody (Roche) diluted 1∶2000 in K block overnight incubation at 4°C. Removal of unbound antibody was performed by washing in maleic acid buffer (100 mM maleic acid, 150 mM NaCl, 0.1% Tween 20, pH 7.5) for 2 hr in six changes of buffer. After washing, specimens were incubated in alkaline phosphatase buffer (100 mM Tris, pH 9.5; 100 mM NaCl; 50 mM MgCl_2_; 0.1% Tween-20 brought up to volume with 10% polyvinylalcohol solution). Hybridization signals were detected by adding 450 µg/mL nitro blue tetrazolium (Roche) and 175 µg/mL 5-bromo-4-chloro-3-indolyl phosphate (Roche) in alkaline phosphatase buffer. After development, worms were washed in PBS then de-stained with 100% ethanol. Worms were mounted in 80% glycerol and imaged using a Zeiss AxioStar Plus microscope. Image analysis was done using ImageJ software.

### Transcriptional analysis of female enriched genes during regression

Adult worms were obtained from infected mice as described above. All parasites were cultured in BME medium [22] supplemented with 0.1% gentamicin (Gibco), 2% fungizone (Fisher), and 1% penicillin/streptomycin (Cellgro) at 37°C/5% CO_2_. Ten unpaired females or 10 male-female pairs were cultured in 5 ml of medium in 6-well tissue culture plates. For seven day cultures, the media was changed every 48 hr. To relax male parasites and facilitate separation of females from males, worm pairs were briefly incubated in 2.5% Tricane (Sigma) in media. Only females that were paired for the entire duration of the study were used for further analysis. cDNA synthesis from mature and *ex vivo* cultured worms was performed as described above. Real time quantitative RT-PCR (qPCR) primers for all genes were designed using Integrated DNA Technologies qRT-PCR primer design software (Table S3). qPCR was done in triplicate using a 7900 HT Fast Real Time PCR System (Applied Biosciences) and EvaGreen Sso Fast master mix (BioRad). Expression changes were quantified using the 2^−ΔΔCt^ method [23] normalized to β-tubulin expression. Dissociation curves were performed for each primer pair to verify specific amplification of one product.

## Results and Discussion

### Gene identification

Nineteen transcripts that were found by SAGE to be present at higher levels in AFMS and two with higher transcript abundance in AFSS were chosen for further analysis. Table 1 lists all genes studied, their SAGE tag counts, their GenBank accession numbers, potential functions or conserved protein domains and literature citations. In this study, the reference ID for each gene is its SAGE tag number. Bioinformatic analysis of these differentially expressed genes demonstrated that they fall into three major categories; genes containing conserved domains that have orthologs with other organisms, trematode specific genes, and genes that do not appear to have orthologs outside of the genus *Schistosoma*.

**Table 1:** Genes investigated in this study.

Gene	Sage tag counts					Genbank Acc. #	Potential function or conserved protein domains	Reference
	AFMS	AFSS	AMMS	AMSS	Liver			
Tyrosinase	52	0	4	0	0	AAP93838	eggshell synthesis	14
p14	1022	3	84	6	1	CAA29285	eggshell structure	13
Fs800	20	1	4	0	0	P16463	TES domain	24
FsMucin	*	*	*	*	*	CAA81792	Mucin	27
CPEB2	*	*	*	*	*	XP_002582188	RNA processing	-
CPEB3	*	*	*	*	*	XP_002574074	RNA processing	-
cgh-1	18	11	8	6	9	XP_002580378	RNA processing	-
10395	483	1	24	3	0	AAA29934	redox defense	25,26
10435	162	0	8	0	0	XP_002576378	unknown	-
10401	136	0	6	0	0	XP_002574767	C-type lectin	-
1610	108	1	5	6	2	XP_002570007	tetraspanin	-
8056	94	0	2	1	0	XP_002576891	β/γ crystallin	-
10548	92	0	0	0	0	XP_002569768	TES domain	-
21110	71	0	5	0	1	XP_002571487	TES domain	-
10617	64	0	5	0	0	XP_002571504	unknown	-
8987	62	0	5	1	0	XP_002571506	unknown	-
11223	52	0	2	0	0	XP_002571502	TES domain	-
10688	51	0	5	0	0	XP_002571488	TES domain	-
10403	38	0	14	0	7	XP_002578571	brain specific membrane anchored protein	-
10763	22	0	1	0	0	XP_002579132	antistasin	-
28488	20	2	2	0	5	XP_002579589	ectonucleotide pyrophosphatase/phosphodiesterase	-
21733	17	1	2	0	0	XP_002576961	RING finger	-
11283	16	2	4	4	2	XP_002571106	unknown	-
33844	14	0	0	0	0	XP_002574668	P/Q rich	-
10927	13	3	3	3	2	XP_002579590	Initiation factor 2	-
11055	12	0	1	0	0	-	RNA processing	-
11088	11	0	0	1	0	XP_002581986	unknown	-
11779	11	1	1	3	1	XP_002581033	diacylglycerol acetyltransferase	-
6767	0	124	1	2	0	CCD61090	MEG	49
15402	0	16	0	0	0	XP_002573676	Claudin family	-

Genes are identified by SAGE tag number or by gene name. Abbreviations used: AFMS, females obtained from mixed sex infections; AFSS, females obtained from female exclusive infections; AMMS, males obtained from mixed sex infections; AMSS, males obtained from male exclusive infections; Liver, immature, liver stage parasites; CBEP, cytoplasmic polyadenylation element binding protein; cgh-1, conserved germinal helicases 1; TES, trematode eggshell synthesis domain; MEG, micro-exon gene. *, no SAGE data available.

**Figure 1:**
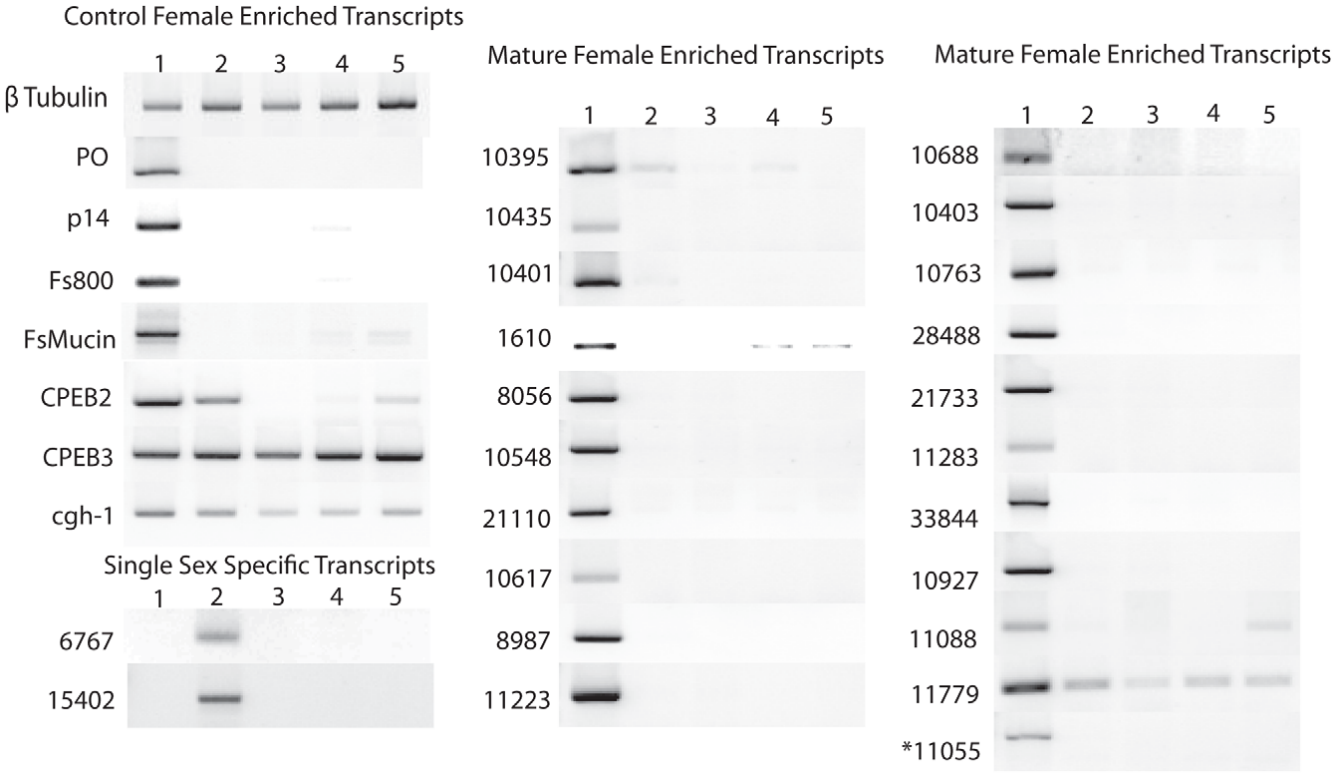
Agarose gel electrophoresis of reverse transcriptase PCR of control transcripts, single sex female specific transcripts, and mature female enriched transcripts. The same cDNA was used for the β-tubulin control and the gene specific PCR reactions. Lanes are: 1: mature female, 2: single sex female, 3: mature male, 4: single sex male, 5: liver stage parasites. All gels shown are normalized to 1 µg of cDNA and β-tubulin controls. The image shown is a composite of multiple agarose gels.

Genes with orthologs in other organisms have a diverse array of conserved domains but they fall into four distinct categories: signaling (11779 and 21733), nucleotide metabolism (10927 and 28488), protein-protein interactions (1610, 10401, and 33844) and mRNA regulation (11055). Trematode specific genes (8056, 10403, 10548, 10688, 10763, 11223, and 21110) have orthologs in other Trematodes (e.g., *Clonorchis sinensis* and *Fasciola hepatica*) but not in any other genera. Proteins encoded by these genes have not been carefully studied and thus the functions predicted by the domain architecture are not confirmed. Four AFMS genes (10435, 10617, 11088, and 11283) contain no conserved domains and appear to be *Schistosoma* specific (orthologs only found in *S. japonicum*). AFSS specific gene 6767 appears to be restricted to the *Schistosoma* spp., while 15402 has orthologs in a diverse array of eukaryotes. RT-PCR was done to verify expression patterns obtained by SAGE. All transcripts had higher abundance in the expected stage (AFMS or AFSS) (Fig. 1).

To better understand female reproductive biology, we included tyrosinase 1, p14 (an eggshell precursor protein), Fs800, a female associated secretory Cu/Zn superoxide dismutase (FsSOD), and a female specific mucin (FsMucin) [13], [14], [24]–[26] as controls. Fs800 and SOD have both been localized to the vitellaria using *in situ* hybridization while FsMucin has been localized to the reproductive duct anterior to the vitellaria using immunohistochemistry [21], [24], [26], [27]. Additionally, p14 and tyrosinase 1 have been localized to the mature vitellocytes using *in situ* hybridization, histochemistry, and immunohistochemistry [14], [21], [28].

SAGE tag 11055 was found to encode a cytoplasmic polyadenylation binding protein (CPEB). CPEBs have not previously been identified or characterized in *S. mansoni*; however, they are known to play important roles in the regulation of mRNA translation in oocytes in other organisms [29]–[31]. Because genomes in other organisms encode up to four CPEB proteins, we queried the *S. mansoni* sequence databases to determine if multiple CPEB proteins were present. We determined that there are two additional CPEB genes. CPEB2 is female specific, expressed in both AFMS and AFSS worms but not other stages examined, while CPEB3 was found to be expressed at similar levels in all life cycle stages examined (Fig. 1).

To better study oocyte maturation we examined the transcription of conserved germline helicase 1 (*cgh-1*), a transcript identified in *Caenorhabditis elegans* that regulates the expression of maternal mRNAs in developing oocytes [32]. In addition, cgh-1 is directly linked to apoptosis in the germline of *C. elegans* and is well conserved across invertebrate and vertebrate species [32], [33]. A recent study demonstrated that there is an increase in apoptosis in the vitellarium of regressed *S. mansoni* females [34]. In *S. mansoni, cgh*-1 mRNA is present at higher levels in females when compared to males and liver stage parasites (Fig. 1), a finding confirmed by mining the SAGE database (Table 1).

### Transcriptional analysis of AFMS specific genes

In order to examine the tissue specific presence of each gene we localized transcripts using WISH. In order to gain a better understanding of the molecular changes that occur as the female reproductive tissues regress to an immature state, we monitored transcript abundance in cultured, *ex vivo* worms using qPCR. We hypothesized that molecular changes occur prior to the visible involution of the vitellarium and the ovary. To examine both early and late events in regression of the female reproductive tissues we cultured *ex vivo* worms for one, two, three, and seven days. Females were cultured both in the presence and absence of male worms to determine if pairing status affected transcript abundance during regression.

**Figure 2:**
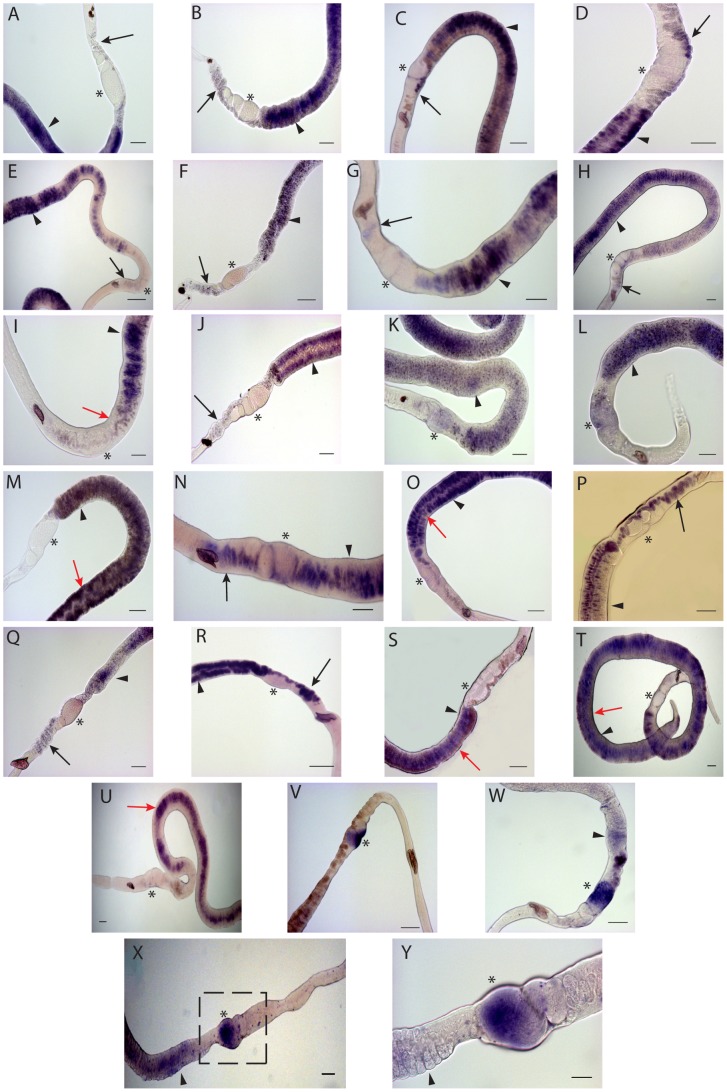
WISH analysis of genes differentially transcribed in mature adult females. Whole-mount *in situ* hybridizations with riboprobes specific to each gene are shown. Panel A: p14; PanelB: FsMucin; Panel C: Fs800; Panel D: 10688; Panel E: 10548; Panel F: 11223; Panel G: 21110; Panel H: 11779; Panel I: 28488; Panel J: 10763; Panel K: 10401; Panel L: 21733; Panel M: 33844; Panel N: 10927; Panel O: 10403; Panel P: 8056; Panel Q: 8987; and Panel R: 10617, Panel S: 10435; Panel T: 11088; Panel U: 11283; Panel V:, 11055/CPEB1; Panel W: CPEB2; Panels X and Y: cgh-1, the boxed region in (X) is shown at higher magnification in (Y). Tissues indicated are vitellarium (arrowhead), the vitelline duct and the ovo-vitelline duct (black arrow), and the ovary (*). All images are representative of >30 female worms. Scale bars are 100 µm for all panels except Y (50 µm). Images were taken on a Zeiss AxioStar Plus and analyzed with ImageJ software.

**Figure 3:**
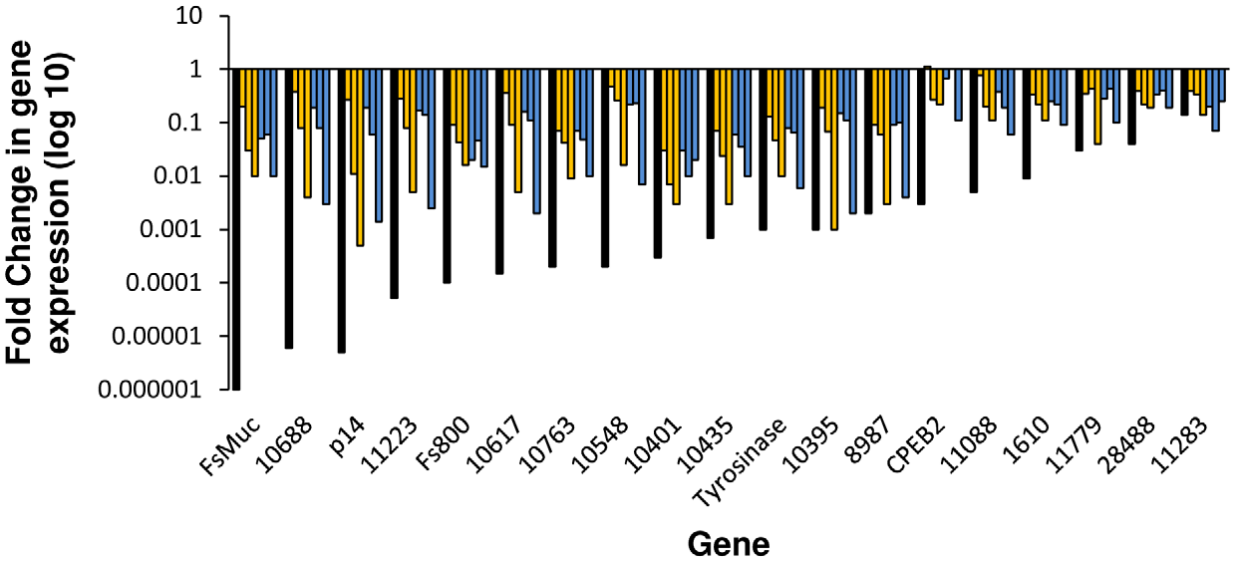
Analysis of transcripts enriched in reproductively mature females in cultured, *ex vivo* worms. Transcript abundance was measured by quantitative real time PCR and analyzed using the 2^−ΔΔCt^ method. Fold changes are measured relative to *S. mansoni* β-tubulin and normalized to uncultured, mature females. Black bars represent relative abundance in females from a single sex infection (AFSS:AFMS). Gold bars show gene expression changes in females that have been cultured with males (from left to right) for 1, 2, 3, and 7 days. Blue bars show gene expression level changes in unpaired females (left to right) at 1, 2, 3, and 7 days in culture.

In a previous study we found that tyrosinase 1 mRNA localized to vitellocytes in the vitellarium, the vitelline duct, and the ovo-vitelline duct [21]. By contrast, p14 transcripts are only detected vitellocytes in the vitellarium and vitelline duct, but not in vitellocytes the anterior ovo-vitelline duct (Fig. 2A). Prior research found that eggshell precursor proteins and tyrosinase 1 are packaged in granules within vitellocytes [7], [13], [14], [35], [36]. Our results suggest that p14 is packaged into granules by maturing vitellocytes prior to reaching the ovo-vitelline duct, while tyrosinase 1 is expressed later. In agreement with prior reports [34], the abundance ofp14 transcripts decreases as female parasites undergo regression regardless of the pairing status of the female (Fig. 3). We also find that tyrosinase 1 exhibits a similar decrease in transcript abundance following *in vitro* regression of the female (Fig. 3).

FsMucin mRNA has a different localization pattern than p14 or tyrosinase 1 and is detected mainly in the vitelline and the ovo-vitelline ducts in the duct epithelium rather than the vitellocytes within the duct (Fig. 2B). It has been suggested that FsMucin acts as a protective barrier to the duct epithelium in the vitelline and ovo-vitelline ducts that prevents premature eggshell formation [27]. Although FsMucin has a different localization pattern than p14 and tyrosinase 1, FsMucin has a similar decrease in transcript abundance following *in vitro* regression of female worms and these decreases in abundance were independent of the presence or absence of a male worm (Fig. 3).

**Figure 4:**
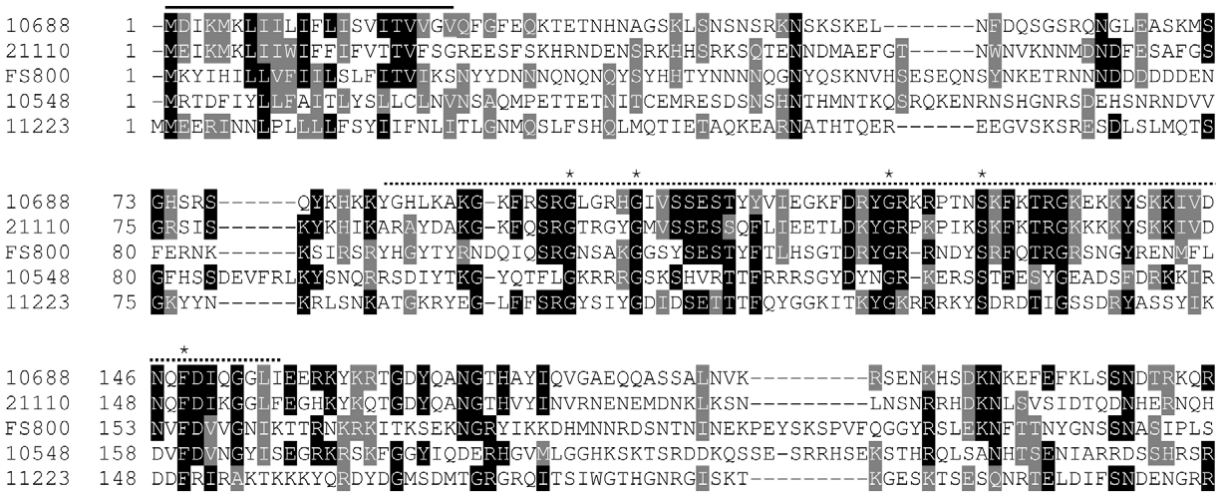
Alignment of TES domain containing proteins. The signal peptide domains are overlined with a solid line, while the TES domain is overlined with a dashed line. Alignments were generated using ClustalW and BoxShade. Residues in black are identical in highlighted proteins while residues in grey are conservative changes.

**Figure 5:**
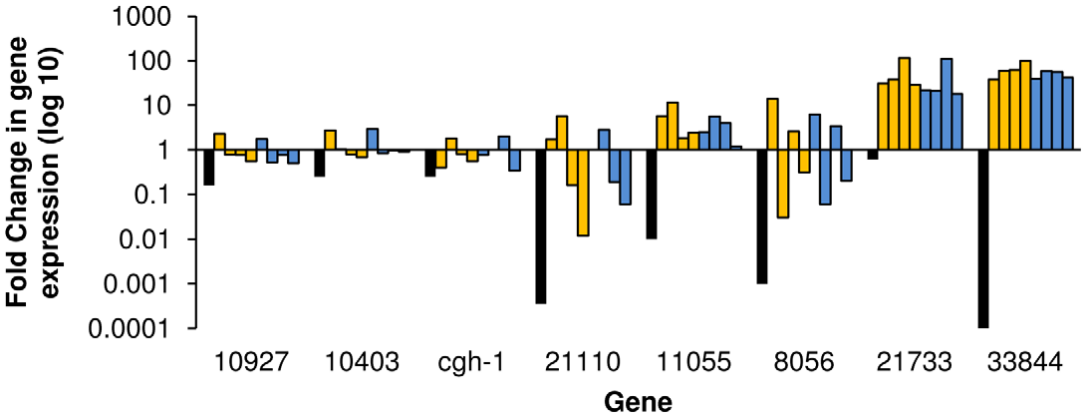
Analysis of transcripts enriched in reproductively mature females during *in vitro* culture. Relative transcript abundance was measured by quantitative real time PCR analyzed using the 2^−ΔΔCt^. Expression of *S. mansoni* β-tubulin was used as an endogenous control and female worms from a mixed sex infection without *in vitro* culture were used for normalization. Black bars represent the relative abundance in unpaired, sexually immature females compared to paired, sexually mature females (AFSS:AFMS). Gold bars represent expression in AFMS females paired with a male after 2, 3, and 7 days (from left to right) in culture. Blue bars represent AFMS females cultured without a male after (from left to right) 2, 3, and 7 days, respectively.

Several of the trematode specific genes (10548, 21110, 11223, and 10688) contain a trematode eggshell synthesis (TES) domain [13], which is also present in Fs800 (Fig. 4) [13], [24]. TES domains are found in a diverse array of trematode species including *F. hepatica*, *C. sinensis*, and *Opisthorchis viverrini* [13]. Proteins with this domain comprise a unique class of eggshell proteins that are found in the eggshell, but are not classified as eggshell precursor proteins from the p14, p19, or p48 families. Unlike eggshell precursor proteins, TES domain containing proteins contain a lower percentage of glycine and tyrosine residues (6.1% Y, 6.5% G, on average) when compared with eggshell precursor proteins p19 (11.6% Y and 9.6% G), p14 (11% Y and 44% G), and p48 (26% Y and 15% G) [37]. Within the TES domain there is a conserved sequence (G-X_4_-G-X_18–20_-G-X_3–6_-S-X_12–19_-F). However, the function of proteins is unknown. Like tyrosinase 1, Fs800 transcripts localize to the vitellarium and vitellocytes in the vitelline duct and the ovo-vitelline duct (Fig. 2C), a finding that confirms results from *in situ* hybridizations on worm sections [24]. The other TES domain containing proteins (10688, 10548, 11223, and 21110) have a similar transcript tissue distribution bearing more similarity to tyrosinase 1 than p14 suggesting that TES proteins are packaged in granules later than the eggshell precursor proteins (Fig. 2D–G). Overall, this indicates that Fs800-like proteins have a different function in eggshell synthesis than p14. With the exception of 21110, all TES genes show decreased transcript abundance following *in vitro* regression (Fig. 3). 21110 shows a transient increased transcript abundance in the first two days of culture followed by a rapid decline in both paired and unpaired females after three in culture (Fig. 5).

Transcripts of several genes with conserved domains found in diverse range of species (10395, 10401, 1610, 10763, 24488, and 11779) localize to different female reproductive structures. 10395, a previously characterized SOD, has a unique localization pattern with transcripts being detected in immature vitellocytes [21]. Because 10395 encodes a Cu/Zn SOD protein and is present in immature vitellocytes, it may be functioning to detoxify superoxide radicals generated early in vitellogenesis due to high metabolic requirements of the rapidly differentiating cells. In *ex vivo* cultured female parasites the abundance of 10395 mRNA decreases (Fig. 3). During regression vitellocytes become less metabolically active and require less SOD activity, a finding supported by the decreased abundance of 10395 transcripts as the vitellarium regresses *in vitro*. However, prior research was not able to biochemically demonstrate SOD function in this protein [25], [26].

11779 transcripts are detected in mature vitellocytes and the vitelline and ovo-vitelline ducts (Fig. 2H). 11779 contains a diacylglycerol acetyltransferase domain (DAGAT) (Fig. S1A). DAGAT domain containing proteins catalyze the terminal step of triacylglycerol formation. In addition, these enzymes maintain steady state levels of the signaling component diacylglycerol [38]. 11779 shows decreased transcript abundance in response to reproductive regression (Fig. 3). Because only mature vitellocytes contain lipid droplets made up of triacylglycerol and other lipids, we hypothesize that the decreased 11779 transcription is reflective of the decreased synthesis of lipid-droplet containing mature vitellocytes.

28488 transcripts localize only to mature vitellocytes in the vitellarium and in the vitelline duct and are not detected in vitellocytes in the ovo-vitelline duct (Fig. 2I). 28488 contains a type I phosphodiesterase/nucleotide pyrophosphatase domain that is similar to human ecto-nucleotide pyrophosphatase/phosphodiesterase-2 (Fig. S1B). In humans and other organisms, these proteins function as phosphodiesterases as well as phospholipases, catalyzing the production of lysophosphatidic acid. The synthesis of lysophosphatidic acid and its subsequent excretion into extracellular fluid induces growth factor responses including cell proliferation [39]. 28488 is predicted contain a signal peptide and to be targeted to the secretory pathway or the cell membrane (Fig. S1B). It may be functioning in the vitellarium to generate lysophosphatidic acid, inducing vitelline cell proliferation, a process that would be required in the vitellarium, but less in the vitelline and ovo-vitelline ducts. 24488 also has decreased transcript abundance as female parasites regress *in vitro* regardless of the pairing status of the female (Fig. 3). Together, these results may indicate that both the maturation and proliferation of vitelline cells are affected by *in vitro* regression of the vitellarium.

10763 has weak similarity (4.48e-05) to antistasin (pfam02822), which can inhibit trypsin family proteases [40] (Fig. S1C). 10763 transcripts are detected in vitellocytes in the vitellarium and in the ovo-vitelline duct (Fig. 2J). 10763 transcripts are decreased in the vitelline duct prior to merging with the ovo-vitelline duct (Fig. 2J). During *in vitro* regression of female parasites the 10763 transcripts decrease in abundance (Fig. 3).

10401 has a unique transcription pattern with mRNA localizing to the vitellocytes both in the vitellarium, in the ducts, and also in the ovary (Fig. 2K). 10401 protein contains a C-type lectin domain (Fig. S1D). C-type lectins play a variety of roles in mediating cell-cell interactions. 10401 transcript abundance rapidly decreases after one day in culture regardless of the pairing status of the female (Fig. 3).

Interestingly, 1610, encoding a tetraspanin protein, has an mRNA localization pattern similar to tyrosinase 1 [21]. Twenty eight tetraspanin genes are present in the *S. mansoni* genome (17), the most well characterized being Tsp2 [41]. These proteins have four transmembrane domains with a large extracellular loop between the 3^rd^ and 4^th^ transmembrane segments. 1610 is a member of the CD63-like tetraspanin group (Fig. S1E). Tetraspanins act as “molecular facilitators” on the cell surface by interacting with laterally located proteins forming a scaffold that can aid in the formation and stability of signaling complexes [42]. 1610 also has decreased mRNA abundance following regression *in vitro* regardless of the pairing status of the female parasite (Fig. 3).

21733 encodes a protein containing a RING (Really Interesting New Gene [43]) finger domain at its carboxyl terminus (RING-variant domain (smart00744) C-X_2_-C-X_10–45_-C-X_1_-C-X_7_-H-X_2_-C-X_11–25_-C-X_2_-C, where X is any amino acid) (Fig. S1F). RING finger domain containing proteins have a conserved Zn binding domain enriched in cysteine and histidine residues. This domain binds two Zn atoms in a ‘cross brace’ arrangement [43]. RING finger domain containing proteins are exclusively found in eukaryotes, and generally interact with E2 ubiquitin conjugating enzymes. 21733 transcripts are detected in the vitellocytes in the vitellarium, the vitelline duct (not in the ovo-vitelline duct), and the posterior ovary (Fig. 2L). A very large increase in 21733 transcript abundance (∼50 fold) during regression may indicate an increase in ubiquitin targeted protein degradation occuring during the involution of reproductive tissues (Fig. 5).

33844 encodes a protein very rich in proline and glutamine residues, with P+Q = 45% of total (Fig. S1G). 33844 is predicted (SecretomeP 2.0, NN-score = 0.858) to be a non-classically secreted protein. 33844 transcripts localize to vitellocytes in the vitellarium and the vitelline duct (Fig. 2M). The transcription of 33844 is greatly increased (∼60 fold) in females undergoing regression (Fig. 5).

10927 encodes an initiation factor 2B subunit (eIF2B) (Fig. S1H). These proteins function in guanine nucleotide exchange between guanine diphosphate to guanine triphosphate (GDP to GTP) to facilitate translation initiation by eIF2B in a number of proteins involved in vitellogenesis [44]. 10927 transcripts are detected in vitellocytes in the vitellarium, vitelline/ovo-vitelline ducts, and the ootype (Fig. 2N). 10927 does not exhibit large changes in mRNA abundance during *ex vivo* culture (Fig. 5).

10403 contains a signal peptide domain (predicted by SignalP) and two transmembrane domains flanking a brain-specific membrane anchored protein domain (pfam12280, E-value: 3.69e-37) (Fig. S1I). This protein domain contains many glycosylation sites and is only found in eukaryotes [45]. 10403 transcripts are detected in all vitellocytes in the vitellarium and vitelline duct but not in the ovo-vitelline duct (Fig. 2O). 10403 shows a transient increase in transcript abundance after one day in culture followed by a return to levels seen in mature female parasites, but not silenced to levels detected in AFSS worms (Fig. 5).

8056 has weak similarity to β/γ crystallin domain containing proteins [46] (pfam00030, E-value: 0.054) (Fig. S1J) and its transcripts are found in vitellocytes within the vitellarium, vitelline duct, and the ovo-vitelline duct (Fig. 2P). 8056 transcript abundance fluctuates during regression (Fig. 5). Further study of chaperone proteins and their functions in vitellogenesis and eggshell formation will allow us to determine the exact functional significance of this unique expression pattern.

Transcripts from several genes that have no significant orthology to proteins outside of the *Schistosoma* genus, 8987, 10617, and 10435, localize to vitellocytes in the vitellarium, the vitelline duct, and the ovo-vitelline duct (Fig. 2Q, R, and S, respectively). 8987 and 10617 are weakly similar in protein sequence to TES domain containing proteins (Fig. S1K). However, they lack the signal peptide sequence and the conserved internal motif (Figure 4). 10435 does not show orthology to any conserved domains after BLAST or PSI-BLAST comparison but has an orthologous sequence in *S. japonicum* (Fig. S1L). Transcript abundance of these genes decreases during regression (Fig. 3).

11088 transcripts are detected in vitellocytes in the vitellarium and the vitelline duct (Fig. 2T), while 11283 transcripts are detected only in the vitelline duct epithelium (Fig. 2U). Both 11088 and 11283 only have orthologs to uncharacterized *S. japonicum* proteins (Fig. S1M and S1N, respectively). Without genome information available for more Trematodes, it remains to be seen if these genes are specific to schistosomes or if they have orthologs in other trematode species that have not yet been sequenced.

### CPEBs play multiple roles in female reproductive biology

Cytoplasmic mRNA polyadenylation is a widely conserved mechanism to control translation, and cytoplasmic polyadenylation element binding proteins (CPEBs) have been shown to function in translational control in oocytes [29], [30], [47]. CPEBs bind a cytoplasmic polyadenylation element (CPE) located in the 3′ UTR of some mRNAs. In *Xenopus*, CPEB is phosphorylated at Ser 174 by Aurora A, a serine/threonine kinase, allowing it to bind other factors that attract poly (A) polymerase to the end of the mRNA thus promoting polyadenylation and translational activation [29]. Without phosphorylation by Aurora A, CPEB remains bound to the CPE, stabilizing the mRNA but not permitting polyadenylation and translation. In this way, oocytes are able to synthesize and store mRNAs that will be required once the oocyte begins to mature [31], [48].

The *S. mansoni* genome encodes three CPEB genes (Table 1 and Fig. S1O). From the SAGE data, CPEB1 (11055) was identified as having increased mRNA abundance in AFMS (Table 1). CPEB1 transcripts localize to the posterior ovary (Fig. 2V). CPEB2 transcripts are detected in both AFMS and AFSS worms (Fig. 1) and localize to the posterior ovary and the vitellarium of AFMS worms (Fig. 2W). These results indicate that these CPEBs are playing different functions in *S. mansoni*. CPEB1/11055 may play a role in oocyte maturation as in *Xenopus* [29], [48]. Based on the transcription of CPEB2 in vitellocytes and its transcription in mature and AFSS worms, we can infer that CPEB2 is functioning in multiple pathways. The large increase in CPEB1/11055 transcript abundance during regression (Fig. 5) may indicate a role in translational repression of oocytes when females undergo reproductive regression. As oocytes cease to mature, there is an increased need to maintain translational repression of mRNA by CPEB. In contrast, CPEB2 transcript abundance generally decreases during *ex vivo* culture (Fig. 3). Further study of CPEB function in *S. mansoni* will be needed to determine the full extent of translational control mediated by CPEB in the life cycle of this parasite.

### cgh-1 controls germline apoptosis in the ovary and vitellarium

Because *cgh-1* mRNA is present at higher levels in mature females than other life cycle stages (Fig. 1 and Table 1) we hypothesized that cgh-1 may be controlling apoptosis in oocytes similar to its function in *C. elegans* (Fig. S1P). In support, *cgh-1* transcripts are detected at high levels in the posterior ovary, where mature oocytes are located (Fig. 2X, Y). Additionally we found that *cgh-1* transcripts are present in vitellocytes, indicating that *cgh-1* may also be controlling apoptosis of vitellocytes (Fig. 2X). When female worms are cultured with a male, *cgh-1* transcripts increase in abundance after two days in culture (Fig. 5), while in female worms cultured without a male an increase occurs after three days in culture. Despite the transient increases in *cgh-1* transcript abundance following regression, we still find overall decreases in *cgh-1* transcript levels by seven days in culture. Increased rates of apoptosis have been shown to occur in the vitellarium within five to seven days in culture [34]. Overall, our results suggest a role for *cgh-1* in maintenance of germline apoptosis in the ovary and the vitellarium.

### AFSS enriched transcripts may function in male-female parasite interactions

**Figure 6:**
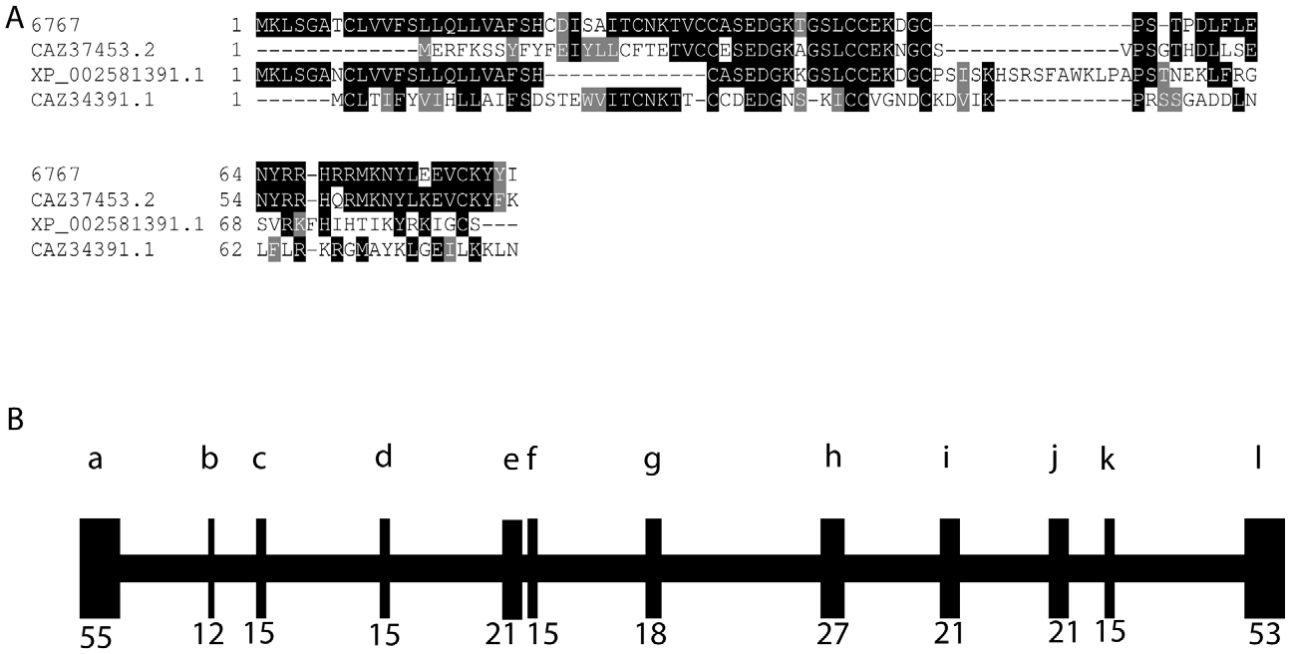
6767 is a MEG-2 Family member. Panel A: Alignment of MEG-2 family members. Proteins include an ESP-15 (CAZ37453.2) and 6767, a MEG-2 family member that is enriched in females from single sex infections. Alignments were generated using ClustalW and Boxshade. Panel B: Exon map of 6767 on *S. mansoni* genome fragment SC_0319. Exons are designated a-l, sizes of exons are listed below exons.

**Figure 7:**
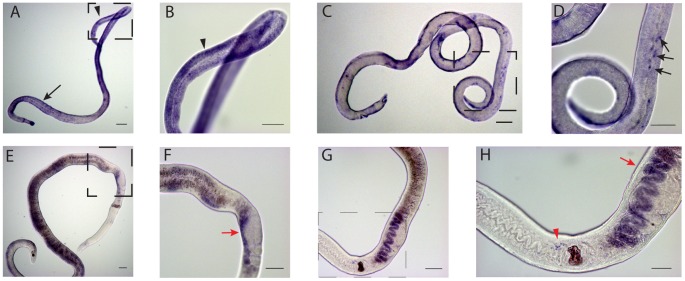
WISH analysis of AFSS transcripts in female worms. Panel A: 6767 localized in AFSS worms. Panel B: higher magnification view of the boxed area in image A. 6767 localizes to vitellocytes (arrowhead) and subtegumental cytons (arrow). Panel C: 15402 localized in AFSS worms. Panel D: higher magnification view of the boxed area in image C. 15402 localizes in a punctate staining pattern in subtegumental cytons (arrows in D). Panel E: 6767 localized in AFMS worms following *in vitro* culture for 3 days. Panel F: higher magnification view of the boxed area in image E. 6767 transcripts are detected in the ovo-vitelline duct (red arrows). Panel G: 15402 localized in AFMS worms following *in vitro* culture for 3 days. Panel H: higher magnification view of the boxed area in image G. 15402 transcripts are detected in the ovo-vitelline duct (red arrows) and in a punctate staining pattern surrounding the ootype (red arrowhead). Scale bars are 100 µm in A,C, E, F and G and 50 µM in B, D, and H. All images generated with a Zeiss AxioStar Plus microscope.

In addition to transcripts enriched in mature females, we also identified two transcripts highly enriched in females obtained from single sex infections; 6767 and 15402. As shown in Figure 1, both 6767 and 15402 transcripts are exclusively present in females from single sex infections, confirming the results of SAGE (Table 1) [18]. 6767 is a micro-exon gene (MEG) (Fig. 6) [17]. These genes are composed of alternatively spliced, very short exons that produce a wide range of variant peptides. MEGs have been localized in juvenile parasites to secretory organelles surrounding the esophageal gland and MEG-2 encoded proteins are secreted by eggs and thought to mediate interactions between the egg and the host immune response [49]. Schistosome MEGs show no homology with genes from other organisms. We mapped 6767 to *S. mansoni* a genomic fragment SC_0319. Other characterized 2.1.i MEGs map to SC_0446. 6767 has the same gene structure as other MEGs of 12 exons with a long N-terminal exon containing 55 amino acids, 10 smaller, symmetrical exons ranging in size from 6–21 amino acids, and a longer C-terminal exon (Fig. 6B). 6767 also has an N-terminal leader sequence and no membrane spanning regions (Fig. 6A). These results indicate that 6767 is most likely a secreted protein, similar to other MEG-2 proteins. In AFSS worms, 6767 transcripts are detected in subtegumental cytons and in stage 1 vitellocytes, the only vitellocytes present in these worms (Fig. 7A, B).

**Figure 8:**
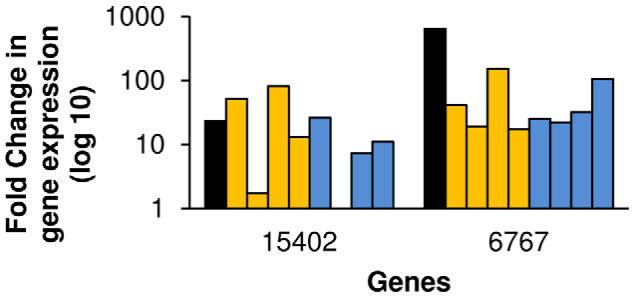
Transcription of sexually immature worm-enriched genes in sexually mature female worms. Relative abundance was measured during regression using the 2^−ΔΔCt^ method. *S. mansoni* β-tubulin was used as an endogenous control and female worms from a mixed sex infection without *in vitro* culture were used for normalization. Black bars represent the relative abundance in unpaired, sexually immature females compared to paired, sexually mature females (AFSS:AFMS). Gold bars represent abundance in females paired with a male after 1, 2, 3, and 7 days (from left to right) in culture. Blue bars represent females cultured without a male after (from left to right) 1, 2, 3, and 7 days, respectively.

Although 6767 transcripts are present at very low levels in AFMS worms, their abundance is increased ∼50 fold when these worms are removed from their host and placed in culture in the presence or absence of a male worm (Fig. 8). Following reproductive regression of AFMS worms, 6767 transcripts are detected in vitellocytes within the ovo-vitelline duct (Fig. 7E, F). Because 6767 is a member of a larger gene family and detection of other MEG transcripts by cross-hybridization may have occurred, we performed WISH on *ex vivo* adult females without *in vitro* culture and were not able to detect any signal (data not shown).

15402 may be a *Schistosoma* claudin gene (Fig. S1Q). The claudin genes encode a family of proteins important in tight junction formation and function [50]. Four transmembrane spanning regions are predicted in the protein encoded by 15402 as in other claudin proteins. In AFSS worms, 15402 transcripts were localized to subtegumental cytons, indicating that the protein product of 15402 may be present in the tegument (Fig. 7 C–D). Like 6767, 15402 gene expression increases (∼20 fold) in response to female reproductive regression (Fig. 8). 15402 transcripts were found in vitellocytes in the ovo-vitelline duct and in cells surrounding the ootype in AFMS worms cultured *in vitro* for three days (Fig. 7 G–H). 15402 expression in mature vitellocytes and in cells surrounding the ootype suggests that it has multiple functions in female reproductive biology.

### Summary

In this study we have characterized genes that are differentially transcribed in *S. mansoni* female worms based on their transcript tissue localization and changes in transcript abundance during regression. Although previous studies have examined female reproductive regression through the lens of egg production [8], [12], [34], [51], [52], this is the first study to catalog previously uncharacterized, female enriched transcripts using a regression model yielding a better understanding of female reproductive biology. In addition, we used bioinformatic approaches to define the conserved domains and protein architecture of these genes; providing us with additional information used to cluster female specific genes. We have shown that many AFMS enriched genes are transcribed in vitellocytes and can be clustered based on their presence late in vitellogenesis (in the ovo-vitelline duct) or at earlier stages (present in vitellarium or posterior vitelline duct). Several genes were found to be transcribed in the ovary exclusively or in the ovary and vitellocytes. We found that the AFMS-enriched transcripts cluster into two groups following *in vitro* regression: 67% of genes have decreased transcript abundance (Fig. 3), while 33% of the genes have increased transcript abundance following regression (Fig. 5). The changes in mRNA abundance observed were independent of male-female pairing. Transcripts with decreased abundance in response to regression localize to the vitellarium, vitellocytes within the vitelline and ovo-vitelline ducts, and the vitelline and ovo-vitelline duct epithelium. Based on protein predictions and localizations, this decrease in abundance may be indicative of their functions in egg biogenesis or synthesis and maintenance of mature vitellocytes. Transcripts that localize to the ovary or the ovary and vitellarium show increased abundance following female reproductive regression. These genes are predicted to encode proteins with diverse functions including oocyte maturation, apoptosis, protein degradation, and vitellocyte-vitellocyte interaction. The genes that are induced by reproductive regression may be essential for maintaining reproductive status in females. Additionally, we examined transcripts that are enriched in AFSS infections. These two genes, 6767 and 15402, may function in the interactions that occur during worm pairing, and may form the basis of an innovative approach to target worm pairing and maturation. Overall, this investigation of *S. mansoni* female reproductive biology illustrates that many genes present in mature females are devoted to diverse processes ranging from nucleotide metabolism to eggshell biology. By using this approach, we can direct research to examine the areas of female biology that are both relevant to understanding the overall process of female development and worm pairing while determining novel therapeutic approaches directed at female schistosomes.

## Supporting Information

Figure S1 **Multiple alignments of genes identified by SAGE.** Alignments were generated using ClustalW and BoxShade servers. Residues in black are identical in highlighted proteins; residues in grey are conservative changes. (A) Alignment of 11779 highlighting the diacylglycerol acetyltransferase domain with orthologs in *Schistosoma japonicum* (AAW27748.1), *Clonorchis sinensis* (GAA40664.2), and humans (NP_079374.2). (B) Alignment of 28488 showing the type I phosphodiesterase/nucleotide pyrophosphatase domain (overlined in black) and orthologs in *S. japonicum* (CAX74907.1), *C. sinensis* (GAA50667.1), and humans (BAA08260.1). (C) Alignment of 10763 showing the antistasin domain. (D) 10401 alignment showing the C-type lectin domain (overlined) with orthologs in *S. japonicum* (AAW25606.1), *C. sinensis* (GAA50649.1), and humans (NP_006030.2). (E) Alignment of the CD63-like tetraspanin 1610 with orthologs in *S. japonicum* (AAW25074.1) and humans (NP_001771.1). The CD63-like domain is overlined and conserved cysteine residues are indicated (*). (F) Alignment of the RING finger domain in 21733 with *S. japonicum* (AAW25925.1), *C. sinensis* (GAA30053.2), and human (NP_848545.1) proteins. Conserved residues are starred (*) and the RING finger domain is overlined. (G) 33844 alignment showing the P/Q rich, putative cell surface domain present in *Aspergillus fumigates* (EDP52841.1) and *S. japonicum* (AAW25874.1). (H) Alignment of 10927 with *S. japonicum* (CAX70293.1), *C. sinensis* (GAA50666.1), and human (NP_001405.1) orthologs showing the translation initiation factor 2 domain. (I) 10403 aligned with orthologs in *S. japonicum* (CAX72358.1), *C. sinensis* (ADZ13680.1), and humans (NP_036241.1) with the brain specific membrane anchored protein domain (pfam12280) overlined in black. (J) 8056 and the orthologs in *S. japonicum* (CAX69522.1), *C. sinensis* (GAA49010.1), and humans (AAA52109.1). The β/γ crystallin domain was identified by PSI-BLAST; one Greek key motif is overlined in black. (K) Alignment of 8987 and 10617 showing weak similarity to Fs800, a trematode eggshell domain containing protein with orthologs in *S. japonicum* (Sjapon8987, CAX70772.1 and Sjapon10617, CAX71239.1). (L) Alignment of 10435 with its ortholog in *S. japonicum* (AAX24261.2). (M) Alignment of 11088 with its ortholog in *S. japonicum* (AAW26070.1). (N) Alignment of 11283 with its ortholog in *S. japonicum* (AAM76790.1). (O) Alignment of *Xenopus laevis* CPEB1 (AAI70313.1) with 11055 (CPEB1), CPEB2, and CPEB3 from *S. mansoni*. The conserved RNA recognition motifs are overlined (RRM1 and RRM2). (P) Alignment of cgh-1 proteins from *S. mansoni* and *Caenorhabditis elegans* (NP_498646.1). (Q) Alignment of 15402 and its ortholog in *S. japonicum* (XP_002573676.1) with the human Claudin protein (NP_001414.1); the transmembrane domains (TM) are overlined in black. Abbreviations: Sjapon, *Schistosoma japonicum*; Csin, *Clonorchis sinensis*; Afumi, *Aspergillus fumigates*; Xlaev, *Xenopus laevis*; Hsapien, *Homo sapiens*.
(DOCX)

Table S1 **Primers designed from SAGE tags for 5′RACE PCR to clone genes differentially expressed in female worms.**
(DOC)

Table S2 **Primers listed were used for cloning genes into pCRII vector for riboprobe synthesis and reverse transcriptase PCR.**
(DOCX)

Table S3 **Primers used for quantitative real time PCR.**
(DOCX)
