# Supplementary material for: Transcriptional Analysis of a Unique Set of Genes Involved in Schistosoma mansoni Female Reproductive Biology
Source: PLoS Negl Trop Dis. 2012 Nov 15;6(11):e1907. doi: 10.1371/journal.pntd.0001907 (PMC3499410; doi:10.1371/journal.pntd.0001907)
Supplement: Figure S1 — Multiple alignments of genes identified by SAGE. Alignments were generated using ClustalW and BoxShade servers. Residues in black are identical in highlighted proteins; residues in grey are conservative changes. (A) Alignment of 11779 highlighting the diacylglycerol acetyltransferase domain with orthologs in Schistosoma japonicum (AAW27748.1), Clonorchis sinensis (GAA40664.2), and humans (NP_079374.2). (B) Alignment of 28488 showing the type I phosphodiesterase/nucleotide pyrophosphatase domain (overlined in black) and orthologs in S. japonicum (CAX74907.1), C. sinensis (GAA50667.1), and humans (BAA08260.1). (C) Alignment of 10763 showing the antistasin domain. (D) 10401 alignment showing the C-type lectin domain (overlined) with orthologs in S. japonicum (AAW25606.1), C. sinensis (GAA50649.1), and humans (NP_006030.2). (E) Alignment of the CD63-like tetraspanin 1610 with orthologs in S. japonicum (AAW25074.1) and humans (NP_001771.1). The CD63-like domain is overlined and conserved cysteine residues are indicated (*). (F) Alignment of the RING finger domain in 21733 with S. japonicum (AAW25925.1), C. sinensis (GAA30053.2), and human (NP_848545.1) proteins. Conserved residues are starred (*) and the RING finger domain is overlined. (G) 33844 alignment showing the P/Q rich, putative cell surface domain present in Aspergillus fumigates (EDP52841.1) and S. japonicum (AAW25874.1). (H) Alignment of 10927 with S. japonicum (CAX70293.1), C. sinensis (GAA50666.1), and human (NP_001405.1) orthologs showing the translation initiation factor 2 domain. (I) 10403 aligned with orthologs in S. japonicum (CAX72358.1), C. sinensis (ADZ13680.1), and humans (NP_036241.1) with the brain specific membrane anchored protein domain (pfam12280) overlined in black. (J) 8056 and the orthologs in S. japonicum (CAX69522.1), C. sinensis (GAA49010.1), and humans (AAA52109.1). The β/γ crystallin domain was identified by PSI-BLAST; one Greek key motif is overlined in black. (K) Alignment of 8987 an [file pntd.0001907.s001.docx]

**A)**

11779 80 FIAPFYRDFLMLFGMIAATKKGLHYLLDKNSCKQTGNFVVVVLGGAPEALDSKPGTYVMHINQRFGFFKLALKTGSYLVP
Sjapon 1 --------------MIAATKKGLHYVLDKDTCKQTGNFVVVVLGGASEALDSRPGTYVMHINQRYGFFKLALQTGSYLVP
Csin 241 FKSPFYRDYLMALGIIAATKRGLRYLLDAEECGKTGNFVVVVVGGAPEALEAKPGQYRLVIRQRYGFFKLAIQTGAYLVP
Hsapien 146 FRAPFFRDYIMSAGLVTSEKESAAHILNR---KGGGNLLGIIVGGAQEALDARPGSFTLLLRNRKGFVRLALTHGAPLVP


11779 160 CISFGEQSLYHQVPNEKGSWIRWLQDKFTSIFTVALPIFYAR-------GPFPYRKPVYTVVGAPIQCEQINEPTDEQVA
Sjapon 67 CISFGEQSLYRQVSNEKGSLIRWLQDKFTSIFTVSLPIFYAR-------GPFPYRKPVYTVVGAPIRCEQIKEPTPEQVA
Csin 321 CVSFGEAGMYKQVRNEPGTILRRLQDWITQLSTFAPPVFYAR-------GPFPYRTPVNTVVGAPIPCERIAEPSQEEVQ
Hsapien 223 IFSFGENDLFDQIPNSSGSWLRYIQNRLQKIMGISLPLFHGRGVFQYSFGLIPYRRPITTVVGKPIEVQKTLHPSEEEVN


11779 233 HIKQIYKEKLRTLFEDYKAIYD-PEANDIEFV
Sjapon 140 NVKQKYIENLQTLFENYKTTYD-PEASNIEFI
Csin 394 HIKQIYLERLRNLFARYKPIFD-PEAHDIEFV
Hsapien 303 QLHQRYIKELCNLFEAHKLKFNIPADQHLEFC

**B)**

28488 33 SS-SRLLVLLIDGLRWDVIAGHLEN-NTNRFGFKRLQKNGAYLQRFTPVFPAECYPNIYSLFTGRHPVDHGVILPTTFSE
Sjapon 33 STTSRLLVLLIDGLRWDVIASHLEN-NTSEFGFKRLQKHGAYLERVTPVFPAECYPNIYSLFTGRHPIDHGVILPTTFNG
Csin 61 HKKSPLLLLLLDSFRWDLLDHFAKRTNHTLRGFNRLITGGGYMKQVRPVFPAECHPNIMSLLTGLYPAEHGMLFTKMYDE
Hsapien 161 FVRPPLIIFSVDGFR----ASYMKKGSKVMPNIEKLRSCGTHSPYMRPVYPTKTFPNLYTLATGLYPESHGIVGNSMYDP

28488 111 HTTINGKPIRSQVEG---LWETGVKQNKGVHLYHLPICSTEAGTENSWYCEPYSQEVMNPMHLNATIQKAVDGLKNGSAN
Sjapon 112 LTTVNNKPIRSRAES---IWETGVKQNKGVHLYHLPICSAETDSESSWYCEPYNQKFMNPIDLNFTIQKAVTGLQNGSAN
Csin 141 RRNRSVGFMEQDVHSPTPDVKTKLAKHGRMHVYHLPFCESLVSEWTS--CEPQEQSQLTPAKLEKTLGRALERLRSGQSD
Hsapien 237 VFDATFHLRGREKFN-----------HRWWGGQPLWITATKQGVKAG---TFFWSVVIPHERRILTILQWLTLPDHERPS

28488 188 LAVVYYDELDRIGHRYGPLSNELVHKHLVYLDHVLDYALNIIESIPNLN---LMLTSDHGMATVS-HQAHADRYFKGTEL
Sjapon 189 LAVVYYDELDRIGHRYGPLSDQLIHKHLVYLDHVVNYMLNIVESIPNLN---ILLTSDHGMSTVS-HEAHADRYLKGTEL
Csin 219 VTVVYYDKLDYVGHEHGPLSDQLLLSHLPALDHVIDRLVDRLTEEAEIRPY-LVVTADHGMTEVLGSTESLDRLFSGHQV
Hsapien 303 VYAFYSEQPDFSGHKYGPFGPEMTN-PLREIDKIVGQLMDGLKQLKLHRCVNVIFVGDHGMEDVTCDRTEFLSNYLTNVD

28488 264 DRVLNRGSTLWIWPKPE-----FENNVYNRLLIQQSKSPFTVYNSSTILSHWEIMTNNNNNNSLLPPVLLIASPNYIFHS
Sjapon 265 SRVLNRGSTLWIWPKPE-----YEANVYNRLLSQQSRSPFTVYNVSTIPSHWEIATTNNNSISLFPPILLIASPDYIFES
Csin 298 KKVINRGSTVSIWPRPE-----EHEKILLRLSSPRIKR-FSTYVNESIPAEWHSGG------ALFPPILLVAKPGFVFNS
Hsapien 382 DITLVPGTLGRIRSKFSNNAKYDPKAIIANLTCKKPDQHFKPYLKQHLPKRLHYANN-----RRIEDIHLLVERRWHVAR

28488 339 KAWPLNMSHYNLIDPKGMHGYDPELPDMHIPLFIYGPNINRGVQLNFTKEIRSVHIHSLMAYLA----------------
Sjapon 340 TVWPLNMSHYNLIKPKGMHGYDPELSDMQIPLFIYGPNSNRGIRLNFTKNIRPIHIHSLIAYLA----------------
Csin 366 EFWPLDLSHTNLSSLKGAHGYDTTHPDMHVPLFLFGPRIKP-SSLYQGIPVDQLYSHQLIASLS----------------
Hsapien 457 KPLDVYKKPSGKCFFQGDHGFDNKVNSMQTVFVGYGPTFKYKTKVPPFENIELYNVMCDLLGLKPAPNNGTHGSLNHLLR

28488 403 -----------SINLPDFRSLDLFKPLLSHNYYHHQLSPVLNNFYWPLNDKSSSSLSFSSTLYGDRFMVNFLIVAAALTC
Sjapon 404 -----------SINLPDFDSLNLFTPLLLP--HHHQPLPSSSYLHWSWNENSSFPL---LSFHGDRFMLSIIIGIVVLSC
Csin 429 -----------TLRP-----------------AFQSSEASSNPVPLQLVDS-------RASVFQNGLFIATLTTIICLLC
Hsapien 537 TNTFRPTMPEEVTRPNYPGIMYLQSDFDLGCTCDDKVEPKNKLDELNKRLHTKGSTEERHLLYGRPAVLYRTRYDILYHT

28488 472 FFVLICAFIILAYVRCRISLTT-TRVFGYQDDLDKELVKSEVLSEA----------------------------------
Sjapon 468 FFVLAIGLLIIVYIRCRFALIMNTRVITCDDELDKELVKSEVLPEA----------------------------------
Csin 474 LVVLLFS----ITFVCRSVLHFCTPLLDVEEKLIQPQGNLA---------------------------------------
Hsapien 617 DFESGYSEIFLMPLWTSYTVSKQAEVSRVPDHLTSCVRPDVRVSPSFSQNCLAYKNDKQMSYGFLFPPYLSSSPEAKYDA

**C)**

* * * * * ** ** *

10763 671 CSSVTCYNQCTQGYEADKYGCPTCKC 696

[Cdd:pfam02822](http://www.ncbi.nlm.nih.gov/entrez/query.fcgi?cmd=Search&doptcmdl=GenPept&db=cdd&term=pfam02822) 1 CPPLKCRKDCPYGFQRDENGCETCEC 26

**D)**

10401 1 MKKFSRITSHILLINLVTWNLLLISSWKISVNNVNPFLTLVYVHGGKNARLSHYDAMSFCRKLAENIQLLNETLIQNATN
Sjapon 1 -------MLLIILINFIIWNILLTSSWKLSFKQISAYITLVYVHGGDDARLSHYDAMIFCQQLPINIKLLNETLIQNATK
Csin 1 -------MSTLPLLLLLSCPLCLYG-LKISEKQFGPYLTLTYVHGGEDAQMSHTQAREFCQNLTS--ELRPEGTTSEAPE
Hsapien 580 FWTALQDLNSTGSFFWLSGDEVMYTHWNRDQPGYSRGGCVALATGSAMGLWEVKNCTSFRARYICRQSLGTPVTPELPGP

10401 81 HN-----------------------ISLQNYINTTLN--AQYYKPLKDYILYGDLVSIHSSTMIHVIMGWVLS------T
Sjapon 74 YN-----------------------ITLNEYMHTTLNESNKSIEQTKQYTLHGDLISIHSPVMIHLIMNWILS------L
Csin 71 NP-----------------------IETRK---------------------RSDLVSIHDPTMVHQLMSWVLP------L
Hsapien 660 DPTPSLTGSCPQGWASDTKLRYCYKVFSSERLQDKKSWVQAQGACQELGAQLLSLASYEEEHFVANMLNKIFGESEPEIH

10401 130 EARQFWIGG---------------LIKLIVHEFN----------------------------------------------
Sjapon 125 EPRQFWIGG---------------LIKLIVHQFN----------------------------------------------
Csin 101 DKRQFWIGG---------------LISKVEHTFQ----------------------------------------------
Hsapien 740 EQHWFWIGLNRRDPRGGQSWRWSDGVGFSYHNFDRSRHDDDDIRGCAVLDLASLQWVAMQCDTQLDWICKIPRGTDVREP

10401 149 GEHRHVIQTWTDHTPVTVRFLHHHS-----------------------------PILERLQ-------------------
Sjapon 144 NEDKHVIQTWTDHTPVTVRFLHHHN-----------------------------NVLENLK-------------------
Csin 120 NQASHVIHTWTDGTPANFRFLHFTH-----------------------------ADFQAFS-------------------
Hsapien 820 DDSPQGRREWLRFQEAEYKFFEHHSTWAQAQRICTWFQAELTSVHSQAELDFLSHNLQKFSRAQEQHWWIGLHTSESDGR

10401 181 ----------------------ANDIACLSIDYASGKWGIHYCTETKYFVCELLKLPKRRT-
Sjapon 176 ----------------------PNDIACLSIDYASGKWGVHYCTERKYFLCELIKIPNRRIK
Csin 152 ----------------------SGATLCLSVDFVSGKWGAHNCKTQMYFVCETINLEEKVP-
Hsapien 900 FRWTDGSIINFISWAPGKPRPVGKDKKCVYMTASREDWGDQRCLTALPYICKRSNVTKETQP

**E)**

1610 1 MASLSCGYKCLQILLVIFNILVFACGIALIVIGSLSQVAINNYSSGIDSNIKGLVIFIIVLGCFLFLLGFLGFCGACTKN
Sjapon 1 MPTLSCGYKCLQFLLITFNILVFACGIALIVIGSLSQVAVNNYSTGIDSNIKGLVIFIIILGCFLFLLGFLGFCGACTKN
Hsapien 1 -MAVEGGMKCVKFLLYVLLLAFCACAVGLIAVGVGAQLVLSQTIIQGATPGSLLPVVIIAVGVFLFLVAFVGCCGACKEN

**
1610 81 TCCLILYAILLSVMVAAEIGAGIAAAVLREDVKTQFLSLVRSSVSEYSKNPDIKKFLDKLQQEFQCCGSESSNDYTSS--
Sjapon 81 TCCLTIYAILLSVMVAAEIGVGIAAAVLKEDVKTSFVSVVRNSVSEYSKNPDIQNLFDKIQTEFKCCGSESSIDYTST--
Hsapien 80 YCLMITFAIFLSLIMLVEVAAAIAGYVFRDKVMSEFNNNFRQQMENYPKNNHTASILDRMQADFKCCGAANYTDWEKIPS

* *
1610 159 --GQTIPDSCKN---------PNTKVTYSDGCSNKVISFFEKYIVAVLVAAFVFAILQLLSIVFAVCVIRAIKSGDSD-
Sjapon 159 --GQSVPSSCTD---------SDTGLAYQEGCSNKIIAFFEKYLIAVVVAAFAFAILQILSIVFTVCIIRAIKSGDTE-
Hsapien 160 MSKNRVPDSCCINVTVGCGINFNEKAIHKEGCVEKIGGWLRKNVLVVAAAALGIAFVEVLGIVFACCLVKSIRSGYEVM

**F)**

* * * * *

21733 321 FYKSTTKSLHKGSLFNLALIVVNNELVKPTKISHTYNPLSNETSLMIFRCRICLDENDHNNETESLLSPCRCKGTVGLVH
Sjapon 1 --------------------------------------------------------------------------------
Csin 202 HSPPTSRS----SPIQTSIFVPKTDRCVPP--DGEVSQQQDDLSFNQFRCRICLDEGELEGP---LMSPCRCKGTVGLVH
Hsapien 70 ------------------------------------------------MCRICHEGSSQE----DLLSPCECTGTLGTIH
 * * *
21733 401 RKCLEKWLLTSGKPNCELCGYAYIMTPSKRHSSQFSTLNQIRRFSNEIRSFRDWLR---WERTRRHLIADIICMILLTPA
Sjapon 1 -------------------------------------------------------------------------MILLTPA
Csin 273 RNCLQRWLYESGKVKCELCGYEYIMTPSRRRS--LPTFTRPRSYT-RLDLFCAWLR---SNTTRRHLMADIICLVLLTPS
Hsapien 98 RSCLEHWLSSSNTSYCELCHFRFAVER-------------------KPRPLVEWLRNPGPQHEKRTLFGDMVCFLFITPL

21733 478 TYIGVYFCVIGAFGYAELNPYSWQVFGLWGLAVLLVLLLTIWMILAIRHHLGNYRSYQHHQQQMALAEANRLSALPRYRF
Sjapon 8 TYIGVYFCAIGALGYAEMNPFAWQVFGLWSLAVILILLLTTWMILAIKHHLSNFRNYQYHQQQIALAEANRLSALPRYRF
Csin 347 TYIGVYFCIVGAMGFAMENPFAWQAIGLWLLAILLIILLTSWMILAIRHHVSTFQRHMYYQRERERLENERFAALPRWRF
Hsapien 159 ATISGWLCLRGAVDHLHFS-SRLEAVGLIALTVALFTIYLFWTLVSFRYHCRLYNEWRRTNQRVILLIPKSVNVP-----

21733 558 SIQPRPRGSSVVLYTIHREQESSPLPKHETQVS-MNSSIESLDIN------SLNNRGVESSCSNKYPNGNQKIVVSVELT
Sjapon 88 SVQPRPRGSSIVHYNVHRDQQSSPLSVNETQIS-LDSNIDNLDVN------SSVSYD-DSSTTNKYTNPNQKIIVSVELT
Csin 427 SIQPRPRGSSLFLRSSTEREATGDLGTLISSESGPPTPVSNLHCSNPHGDFEFQAHIIPQGSSEHSGPISPKFVVSVALS
Hsapien 233 SNQPSLLG----LHSVKRNSKETVV-------------------------------------------------------

21733 631 TVPEVAEEMSTSNNKVSFDNIV
Sjapon 160 TVPEVVEEMSTLNNRLSNENIV
Csin 507 TVPEVTEEYTHPSTEHGSPNMK
Hsapien ----------------------

**G)**

33844 52 ----------------------HYATG-------PSMIPQQPIIPQQPIIPQQPIIPQQPVIP-----QQPVIPQQPVIP
Sjapon 151 KQRKQTDNSMRYISKSYKKSKLLYGTGFMPTLSVPVSIPVQAQVPVSVPVAQPYSIGGHAYMPGHFHYGFPVQPMQPVMP
Afumi 161 VPTGVPGQNTTVVPPP--RPAPFTNSTVPPPVQPVPVAPGQPTAPSQPSVPGQPSVPGQPSVP-----GQPSVPGQPSV

33844 98 -QQP---AIPQQPTPIAPQLPPTVPQQQPPAATPQQP--PEVSPQQPPAATPQQPP-AVSPQQPPAVTPQKPE-IP----
Sjapon 231 PQQPPPMMPPQVPTMVPPQQPPMVPPQQPPVIPPQQP--PAVTPQQPPAETPQQPPTSVTPQQPPAVTPQQPPGIPTQT-
Afumi 234 -GQPSVPGQPSVPGQPSVPGQPSAPGQPSAPGQPSVPGQPSVPGQPSVPGQPSVPGQPSAPGQPSAPGQPSVPGQPSVPG

33844 166 ------FPF-PSPSVKQTQV----QSQIPLQPKVSSVSQMQQLQLLQQQQQLLQTQQQKPQVTVKHVIMPQHIKVFIKSP
Sjapon 308 QPVAPTLPLSPTQPVLQSQT----QTQLSIQPQIPQVPQIPQLPLTP-QHQLIQLQQPK-ALTVKHVILPQQIYLTVKST
Afumi 313 QPSVPGQPSVPGQPSVPGQPSVPGQPSAPEQPSVPGQPSVPEQPSAPEQPSVPGQPTAVSPPPAETALTGSASQFEPAAG

33844 235 NVRPRFIKRYYY
Sjapon 382 RVKPRFIKRIYY
Afumi 393 LLAGIWVVMLML

**H)**

10927 1 -------MTSQILDLVRRFKVLCERNPDKSEAVVAIEVLAQLHECSDVTTVQGLHDVMNAAIERMWKEGVSNLSVISACE
Sjapon 1 ------MTTCQIQDLVRQFEVLCQENPDKSEAVVAIEVLAKLHECSDVTTVQGLYDVMNATIERMWKEGVSNLSVISACE
Csin 1 MYQSLFSKMHSSGDIAGLFRRICKASPEKSEALAAIDVLTRVLHDSHVSTVQGLHDVLNTAILEMTEADHTYLCVKSACE
Hsapien 1 ---------MDDKELIEYFKSQMKEDPDMASAVAAIRTLLEFLKRDKGETIQGLRANLTSAIETLCGVDSS-VAVSSGGE

10927 74 LFQRFITLATLDTVD-FDECKR------------VGSCRRKIAENFLNYIPNGSVIFLHSYSRVVLAALE-YAAS-----
Sjapon 75 LFQRFITLTTLDTVS-FDECKRVLSERAQIFIRKVGSCRRKIAENFLNYIPNGSVIFLHSYSRVVLAALA-YAAS-----
Csin 81 LFQRFITLTISDAPDDFNRCKQVLQERANMFLSKIGACKRQIAENFLNLLPDGGCILVHSYSRVVLSALEAYAASKSNTA
Hsapien 71 LFLRFISLASLEYSD-YSKCKKIMIERGELFLRRISLSRNKIADLCHTFIKDGATILTHAYSRVVLRVLEAAVAA-----

10927 135 -----TQKRLHCYVTTCAPSGLGAKMTKALAKLKVSCTLVPDNSLAYLMPQVDLVVLGAEAVVESGGILNMLGSSLMAMT
Sjapon 148 -----AHKRLHCYVTTCSPNGLGAKMTKALAKLKISCTLVPDNSLAYLMPQVDFVVLGAEAVVESGGILNILGSSLIAMT
Csin 161 ATSNLSSNRLHCYVTTCAPHESGRKMTKELARLKIPCTLIPDLSIGYLMPRVNLVLMGAHAVVESGGVLNDLGSSTVAMI
Hsapien 145 ------KKRFSVYVTESQPDLSGKKMAKALCHLNVPVTVVLDAAVGYIMEKADLVIVGAEGVVENGGIINKIGTNQMAVC

10927 210 ASSFGRPVYVLAESFKFMRCYPLDQRHIPDEFKWSYDHECGSLS-PSALLILGDGNN--YLTQD----ENNCDFPEMKTA
Sjapon 223 ASSLGKPVYVLAESFKFMRCYPLDQRHIPDEFKWSSDHEYKSLSSPSPLFNLRDKSNCYYFPNDDDDDENKCVFPEMKTV
Csin 241 AAAFGKPVYVLAESFKFIRAFPLDQHHIPSELKYSS----GVSPACSPLRNLKMTDT---LEEDE-VEDAEEDFPELHTA
Hsapien 219 AKAQNKPFYVVAESFKFVRLFPLNQQDVPDKFKYKAD-------------------------------------------

10927 283 WADVEAQRVPVIEQKMPRVDYTSPCYINYLITDLGVLTPSAVSDELIKLYL
Sjapon 303 WAAVEAQRVSVIEQKMPRVDYTSPCYINYLITDLGVLTPSAVSDELIKLYL
Csin 313 WARVNEHRVQTIERTMPITDYTNPFYISYLVTDLGVLTPSVVSDELIKLYL
Hsapien 256 -TLKVAQTGQDLKEEHPWVDYTAPSLITLLFTDLGVLTPSAVSDELIKLYL

**I)**

10403 1 ---MRVLVVPLFALLYSLVDAIELPL-----------TPKTCLDVCVTKFDGAE-------------------EDACSRG
Sjapon 1 ---MDIFAVTFLVFFHSLVAAIEFPL-----------TPETCKEVCVSQFDGAE-------------------EDACSRG
Csin 1 --------MVLFLLLFLVVSASSS-------------RVDDCTNSCTASPK----------------------QDACIQG
Hsapien 1 MAAVALMPPPLLLLLLLASPPAASAPSARDPFAPQLGDTQNCQLRCRDRDLGPQPSQAGLEGASESPYDRAVLISACERG

10403 48 CVLSNLNSLPA--------TFTCEESCDDVYKPDTKLVAACKVGCGATPSHTS------IRLGFLPVQNFFSSFFDSIRH
Sjapon 48 CILSNLNSLPN--------TYTCEGSCEDAYMPNNKSVAACKVGCGATPGHTS------ISLGFLPVQNFFGSFFDKFRQ
Csin 38 CYYAYALTLTPEEPK---LRPTCKKSCEENLTGEDE--KACETGCEQFETSD----------GWFGALGLMDTLRDFVSQ
Hsapien 81 CRLFSICRFVARSSKPNATQTECEAACVEAYVKEAEQ-QACSHGCWSQPAEPEPEQKRKVLEAPSGALSLLDLFSTLCND

10403 114 LLGGVNNGSGANADTKTNKVDGDMDSDSGNHVVISRVRIFHNFAPYDAHESPLDSPFDIKPLFSLRGIIPQDDDAYVKAV
Sjapon 114 LLGGVNSGPEVSLNPTANKVDSDSDSNSRNHVVISRIRIFHVFNPFDARENPLES--------SYNEMIPQDGDSYSKSL
Csin 103 MLSGKPD--------DTAIANSELNSEP---VVVSRIRIFIPIEEADLAPEDAPS-----------HALVQPNEKAGSQS
Hsapien 160 LVNSAQG-------FVSSTWTYYLQTDNGKVVVFQTQPIVESLGFQGGRLQRVEVT--------WRGSHPEALEVHVDPV

10403 194 KNSDQNIIRIHAVSGDGESTFHEHQPTFVRKATYFFRHIMFRPLLLPLLIALLIVILLLMVKLTIHACR--------VRE
Sjapon 186 KNDGRNIIRIHSISDEGESTFAEHQPTSVGKVAFFFRHIMFRPLFYTLLIALLIVILLLMVKLTIHACR--------VRE
Csin 161 DQVPHHMMFAHQAPVNSKSVYSGEG------VECWFRRMLHAPFLLLFVVTLSTLVVLLFVQLVLCVRRR-------YSA
Hsapien 225 GPLDKVRKAKIRVKTSSKAKVESEEPQDNDFLSCMSRRSGLPRWILACCLFLSVLVMLWLSCSTLVTAPGQHLKFQPLTL

10403 266 HRHIEYARLPTYIEAMNVKVPLYEDVIKCEKSSEKGPIDA
Sjapon 258 HR--QYIRLPTCIEAMDGKVPLYEDVVKCEKPCEKAAMGV
Csin 228 PRGYRYAPLPTYAEATNIKVPIVGDADELKVPLKQEDA--
Hsapien 305 EQHKGFMMEPDWPLYPPPSHACEDSLPPYKLKLDLTKL--

**J)**

8056 1 MFYFKMVDAMRLLNLLLVSCPTVYALESNHRTTVANNNASELRSSSNLLNWRNTISQCVRLYTGPNELGDWFDICDSNEL
Sjapon 1 -----MLFILPLFVTLLLPG-FTWTLGGNIVTTESNSSSSESNSSSKLLISEYTQLKCLRLYSGPNRVGDWFDICDSNEL
Csin 1 -----MFYLFEFYCIIFTAIAVSLPLESSSSSGLLFWSAEQPK--LLKTSKRHTKLVCLRLYTQRKFQGTWSDICSSNEV
Hsapien 1 ----------------MGKITFYEDRAFQGRSYECTTDCPNLQPYFSRCNSIRVESGCWMIYERPNYQGHQYFLRRGEYP


8056 81 LSSMFVWRAQSMCTSGNTNPIRTIYWL-IYERSHFTGPYILLGPSRCIDDIRQYKFLATTSILICVEIVRTKLDVLVSCQ
Sjapon 75 LSSMYVWRTRSLCAAGNALSHMQTYWL-IYERPHFRGPYLLLGPTRCINDVNQYKLLATTSVLMCTETTRHHLDVSVICH
Csin 74 LLVQSIFNTRSVCAPQGKWPDSNNYWL-LFERPYFIGSYVMLAPNDCVRKL-QSSLSTVGSVLKCTSSG-----LKISCL
Hsapien 65 DYQQWMGLSDSIRSCCLIPPHSGAYRMKIYDRDELRGQMSELT-DDCLSVQDRFHLTEIHSLNVLEGSW-------ILYE


8056 160 YPKQPWRQFFQVNWHSDNKVTSKQSVTVGMKSADEVKASSMENMHSLTELQKNSINNK
Sjapon 154 YPPRPWREFFPI--YSNQGIDTVQYKSVTGDQNALVNPS-----GSLTDLLRNSVNNK
Csin 147 YPPKPWLGFGSFG-QTELEYQAQEVESVKSEIGASWPR--------------------
Hsapien 137 MPNYRGRQYLLRPGEYRRFLDWGAPNAKVGSLRRVMDLY-------------------

**K)**

8987 1 MLLIYFYLITCISPIYSHERYSYDDHDDFEGSFFDDDYGSHGGYHSGNYDNHPQHP--PAHNSPGEGEPSPPYPDDDQEP
Sjapon8987 1 MRLICLCIIIYTPLIYCFRQGLYDYDDD---SFFNDRYDFSSHDYSESYDSDYQSPDKSPNQHHSYDEPDSPLHPNKNES
Fs800 1 MKYIHILLVFIILSLFITVIKSNYYDNN---NQNQNQYSYHHTYNNNNQGNYQSKNVHSESEQNSYNKETRNNNDDDDD-


8987 79 PRAPPEKPEAPHEPDGDENYGRPHYN-HSRYHGYYDDLDKYDSELRELVLSGHRYKPRYYEGEGYGLVGEETYVKKPKDP
Sjapon8987 78 GGLFNYHKNSSHR---HENYKHNHR--KSKYHRYNDDWHRYDDELKDIIFGDGYQQVFSQYTEGHGLLGDETYGSTHRDP
Fs800 77 ----------------DENFERNKKSIRSRYHGYTYRNDQIQSRGNSAKGGSYSESTYFTLHSGTDRYGRRNDYSRFQTR


8987 158 SKYDQYGN--YIPDYQTPQPSPPPPHPTPIPSNSNEQPSGNVGENVNNEEGEDNKPDETEENEYKPTRYVKYKKYKP---
Sjapon8987 153 SKYDRYGN--YMPDYTTPNGSPNPP-----PSSDEGEEEGDEEE--EEEEGDYGGEESETEVVMKPAPYVKYKQYSPQPT
Fs800 141 GRSNGYRENMFLNVFDVVGN---------IKTTRNKRKITKSEKNGRYIKKDHMNNRDSNTNINEKPEYSKSPVFQG---


8987 233 --SNKRVSSKTKSSKKGTKGTKGTKGTKGKRGK
Sjapon8987 224 PIPNTQNMAALKSSKKKKKKKKKSKKKRYYYAK
Fs800 209 ---GYRSLEKNFTTNYGNSSNASIPLS------

10617 1 --------------------------------------------------------------------MDQSKKDSYEAI
Sjapon10617 1 --MLFNQCYSIVLVFYINFITS-----------VFTYYDDYEYG-----------------------MTGEAGRDAEVAL
Fs800 1 MKYIHILLVFIILSLFITVIKSNYYDNNNQNQNQYSYHHTYNNNNQGNYQSKNVHSESEQNSYNKETRNNNDDDDDDENF


10617 13 NFPLKTIHGHKYDMDYGNGFIPNN--YGNHDNHDDQEYDSYHSNDYDDGEEHNYGHVNLAEQS-----------------
Sjapon10617 45 HYTVGKFDGKKYGENY--EYLQDN--YGKYGDYDDYGYDSYYG-----DEGYDYNHVTLADRA-----------------
Fs800 81 ERNKKSIRSRYHGYTYRNDQIQSRGNSAKGGSYSESTYFTLHSGTDRYGRRNDYSRFQTRGRSNGYRENMFLNVFDVVGN


10617 74 ---MRKLKELKHRKYNEKYKGDNEEE-----EEGGEYNDRYYRPRDEPDIESTEETTEGPRRTTTTPIFMGE
Sjapon10617 99 ---MRKLKDMKRRKHHYLSKDYDEGYGYGYGPEVEEYKEPGYNGNNYPGHKPPMLLPPTTKPPSQNQNFRAE
Fs800 161 IKTTRNKRKITKSEKNGRYIKKDHMNNRDSNTNINEKPEYSKSPVFQGGYRSLEKNFTTNYGNSSNASIPLS

**L)**

10435 1 ------MLNKKFIFIFISLFLLNEISYGYILVCRKMKKDSKRSYHGYGGYCPDCPLICRVFTNDMKRPESFQPRKLRPNT
Sjapon 1 IYLISVMLNKNIILLIISLLLLNEISFGYILVCRKMMK--KRYGYNFHGDCPECPLICRVLSNDMSRPTSFKRPKIGPQT


10435 75 CR
Sjapon 79 PR

**M)**

11088 1 MCCCEYDIIKYILIYSITIMMVIYLVSGEDYLITNEDLSKLTGSTTTQTTIDPILLESYRKNTIKSFIIFGVIFGLINIA
Sjapon 1 MCCCEYELMKYILIYLMTLITVIYLVSGQDHLSINEDISRLTDATMQTTTIDPALLESYRKKTIISFTVFGVIFGLMNVV

11088 81 CWIASIIKAIIDRRKIKKRNLARQDAKRFKTESHRKSVLKDLIDPEPPKLILPSIVDSAVKNYKNVSPTTPNLTDSPIHH
Sjapon 81 CWIASIIKAVVDRRRIKRRNLARHEAKRFKSESHRRSVLKDLLEPEPPKLILPNVVDNVIRNYKTDSSTTPTSSESPIHH

11088 161 FSRCSTLRRSDSLKRSTQGEIDEPTEKDLFVK
Sjapon 161 FSRYSTLQRSSSPKQYTQDEMNEPTEKDYFMK

**N)**

11283 1 MSIFRLLCFLITVIMAICTLGVGLILSPYWKGNDEPPREAKIHLSSSLISILFLGASSILIIVSIVSGPSRTKKMIIIVM
Sjapon 1 MSIFGLSCFFLPLLLTVCTLAVGLFLSPYWKENREAPKEAKIHLSLSIISILLLGASSILTLVSIISGPGRTKKIIIIVI

11283 81 ILLILTIGFLCASVVYMFTQINHSFAFWIFSSLWISVTCIPFGLCTLYIQPIQMNRL
Sjapon 81 ILLVITVGFLCASIVYMFTQVNHSFAYWIFASLWISITCIPFGLCTIYIQPIQMERL

**O)**

11055 77 HWSGKLPIRVHRSMT----------------------------------FSRKVFLGGVPWDS-----------------
CPEB2 135 HPPITYHLEVHLTSSKSIYFNQLSNQSMNCFPSVKHHHHHHFNSSFMNRLSCKVFVGGVPMRGN----------------
XlaevCPEB1 262 WPSWDLLDSAEDPFSIEREARLHRQAAAVNEATCTWSGQLPPRNYKNPVYSCKVFLGGVPWDI-----------------
CPEB3 321 KLGSSLVTDSNSPTVCDFPGNEGNTVSWSQNSSVHQTLHKSNVQNIPNMVGPVGFGGGMANGSLNRAAVSVGLVSVSTPG

**RRM-1**

11055 106 ---------TSEELVRAFSRFG-----NVSVCWPQKEGSSSTSTHIKASSN-----------KGYCYLVFEHEVSVTELL
CPEB2 199 ----QVTTWTRDQLHKGLSIFG-----PVSLVWPKG------TIVIPSVID-----------GRYCYAVFNDQRSVSLLL
XlaevCPEB1 325 ---------TETGLINTFRVFG-----ALSVEWPGKDGK-HPRCPPKGNMP-----------KGYVYLVFESEKSVRALL
CPEB3 401 VSITSNNKVNEAGLSNIYSMFPKRPQHRMMAHWQQQQQHHQNSQQVPLGKSPMNIVTSNIQLNGNRGSLTAVNSTLSALP


11055 161 ANCVHNPTT---GGEYYTISSPKFIS---KDVQVIPWVISDS----QYTKSTPSSS-----------------DIKRTVF
CPEB2 253 SMCYRRTKG---FYINLSTLCPDLEKTRFDSLQIIPWDKQDS----FYPILPNRNSGNINFHSEEGRVETETRTKVYSIF
XlaevCPEB1 379 QACSQDLLSQ--DGLSEHYFKMSSRRMRCKEVQVIPWVLADS----NFVRSPSQRLD-----------------PSKTVF
CPEB3 481 NGCEDSGVNNSAGSFNNHSIIQSLNSVGSSSSANSPLVLCSSGDSFTNTLSFNSPTSESSLDSFKYGLDQQLMEVIKSFE

**RRM-2**

11055 214 VGALHA--LITAEILVTIMNDLFGNVIFAALDTDKYKYP-------------------------IGSGRVIFSSHKSY--
CPEB2 326 VGALHG--MITARALFTIFNDLFSNVSYAALDIDKYHYP-------------------------IGSGRVAFNSLEGY--
XlaevCPEB1 436 VGALHG--MLNAEALASIMNDLFGGVVYAGIDTDKHKYP-------------------------IGSGRVTFNNQRSY--
CPEB3 561 ISSIGGSNLASSNANSTSMGGIDDLTLSSSFGEIDQQIPGLNSASQMPVLGSPSIHNSTPMINGSNNQSSSFNSLSSYNG


11055 265 --------MKAITANFVDIRTSK--FIKTIQID---PYLEDAVCNSCLSYPG-------------MYFCRAFECFNYFCP
CPEB2 377 --------LAAIKTNFICIECQL--FSKVIQID---PYLEDAMCSKCFSVPG-------------IYFCRHLKCFDYFCP
XlaevCPEB1 487 --------LKAVSAAFVEIKTAK--FTKKVQID---PYLEDSVCQVCNAQPG-------------PFFCRDQVCFKYFCR
CPEB3 641 SSIMGSAGMTSPYSMFANIFTREEGFSRKVFVGGLPPDIDEEEITTAFRRFGPLIVDWPHKTESKAYFPPKGYCFLLFQD


11055 319 --------------ACWHMWHNSTETLYTHKPLRRTFKPNIDRQWQTGVVVTTTVTTTTATATTTVSVATTTQSRF--
CPEB2 431 --------------KCWIHYHGSKN--TTHKPLRRTLIPNSLKFAKYF------------------------------
XlaevCPEB1 541 --------------SCWH-WQHSMEILRHHRPLMRNQKSRDSS-----------------------------------
CPEB3 721 ERSVQSLINACIVDEGKYYWCVSSPTMKDKPVQIRPWNLADSDFVMDGSQPLDPRKTIFVGGVPRPLRASKLTNKLVV

**P)**

cgh-1 520 DNTANDDWKSTLKLPEKDMRIKTADVTALKGSSFEDFCLKRDILKGIYEKGWESPSPIQESSIPIALTHRDIMARAKNGT
Celegans 12 ANNGDENWKAGLNLPAKDRRFKTADVTDTKGVEFEDFCLGRDLLMGIFEKGWEKPSPIQEASIGVALTGQDILARAKNGT


cgh-1 601 GKTGAYSVPVLESIDTTINKIQAIILVPTRELALQTSQICIELAKHTAIKIMLVIGGTLLKDDLIRLSQTVHVLIGTPGR
Celegans 94 GKTGAYCIPVIEKIQPALKAIQAMVIVPTRELALQTSQICVELSKHIQLKVMVTTGGTDLRDDIMRLNGTVHLVIATPGR


cgh-1 681 LVDLLSRGLIDISKCKIVVLDEADKLLSEELISGVEEILNGVDKSRQVLVYSATYPVTVQSFMNQHLRNPYQINLMETLT
Celegans 174 ILDLMEKGVAKMEHCKTLVLDEADKLLSQDFQGILDRLINFLPKERQVMLYSATFPNTVTSFMQKHMHKPYEINLMEELT


cgh-1 761 LKGITEYYAYVQEKHKVHCLNTLFSKLQISQSIIFCSSAQRVELLAKKITQLGYSCYYIHARMSQQDRNRVFHDFRNGCC
Celegans 255 LLGVTQYYAFVQEKQKVHCLNTLFRKLQINQSIIFCNSTQRVELLAKKITEIGYSCYYIHSKMAQNHRNRVFHDFRQGNC


cgh-1 841 RNLVCTDLLTRGIDIPTVNVVINFDFPKYSETYLHRIGRSGRFGHLGIAINLVTYADRYSLKTVETELVTEIKPIPKEID
Celegans 336 RNLVCSDLLTRGIDIQAVNVVINFDFPRNAETYLHRIGRSGRFGHLGVAINLITYEDRHTLRRIEQELRTRIEPIPKTVD


cgh-1 921 KRLYVAEYQNENNLDPMTR
Celegans 417 PKLYVADQQLVDAADETTA

**Q)**

**TM-1 TM-2**

15402 1 MGESRSVKLFILCGLAIAICFTVVA--IAEDKTLTSEY------------------KSNSLKAFQAFYFLALIAFIVALV

Sjapon 1 MELSRSTKIFTFIGLAILLSFTLTA--MIQDDTLTAQN------------------KSHATKTFQAFYFIAFFSFIAALI

Hsapien 1 MLVLLAGIFVVHIATVIMLFVSTIANVWLVSNTVDASVGLWKNCTNISCSDSLSYASEDALKTVQAFMILSIIFCVIALL

**TM-3 TM-4**

15402 56 LYLVTIFTASRRVLSIGFFVAVVVGCICCIISVIIYYQHMSNSYNRSFRPDSTSWLLVVIVSSAQLILFVCLYILY

Sjapon 56 LYLVIIFVKDLRLLKFGFVGALVVGCFCSIISVIMYYDNLSRYYNATIRPDTSSWLLVVIVSAFQLCLFAFMYILY

Hsapien 81 VFVFQLFTMEKGNRFFLSGATTLVCWLCILVGVSIYTSHYANRDGTQYHHG-YSYILGWICFCFSFIIGVLYLVLRKK
