# Supplementary material for: Transcriptional Analysis of a Unique Set of Genes Involved in Schistosoma mansoni Female Reproductive Biology
Source: PLoS Negl Trop Dis. 2012 Nov 15;6(11):e1907. doi: 10.1371/journal.pntd.0001907 (PMC3499410; doi:10.1371/journal.pntd.0001907)
Supplement: Table S1 — Primers designed from SAGE tags for 5′RACE PCR to clone genes differentially expressed in female worms. (DOC) [file pntd.0001907.s002.doc]

Supplementary Table S1: Primers designed from SAGE tags for 5’RACE PCR to clone genes differentially expressed in female worms. All primers are shown 5’-3’ and used with T3 (5’ TTAATTGGGAGTGATTTCCC 3’) to amplify genes identified by SAGE from the *Schistosoma mansoni* phage λ cDNA library.

| Gene ID | SAGE Tag Primer |
| --- | --- |
| 10435 | AGTATTTATCATTTGTTCATG |
| 10401 | ATTTTTGTTCTTTACAACATG |
| 8056 | AGTTCTGTCAAACTATGCATG |
| 10548 | ATTTACCATCACTAATTCATG |
| 21110 | CAATATTACAAGATTTACATG |
| 10617 | ATCGTACAACAAATATTCATG |
| 8987 | ATATAAAGATCATTTTTCATG |
| 11223 | CGATATCCCACGATTTCCATG |
| 10688 | ATAAATTATAATTTTAACATG |
| 10403 | GTTTACTCCAATGCATTCATG |
| 10763 | AAGATGTTAAGCATTTGCATG |
| 28488 | TGGTGAAGTAGAAGATACATG |
| 21733 | TCGGATACTTATTGGAACATG |
| 11283 | TGAAATGAAAACGTTCACATG |
| 33844 | ATAATTATTATTCTTATCATG |
| 10927 | TTTTTTCGGTGTATGAACATG |
| 11088 | AACACCTTTCTGTTAAACATG |
| 11779 | CCTGTAACCATCGTATCCATG |
| 11055 | AACTATGGGCTCACAAACATG |
