# Supplementary material for: Transcriptional Analysis of a Unique Set of Genes Involved in Schistosoma mansoni Female Reproductive Biology
Source: PLoS Negl Trop Dis. 2012 Nov 15;6(11):e1907. doi: 10.1371/journal.pntd.0001907 (PMC3499410; doi:10.1371/journal.pntd.0001907)
Supplement: Table S2 — Primers listed were used for cloning genes into pCRII vector for riboprobe synthesis and reverse transcriptase PCR. (DOCX) [file pntd.0001907.s003.docx]

Supplementary Table S2: Primers listed were used for cloning genes into pCRII vector for riboprobe synthesis and reverse transcriptase PCR. The annealing temperatures and number of cycles used to generate the gels shown in Figure 1 are listed.

| Gene ID | Forward primer (5’ - 3’) | Reverse primer (5’ - 3’) | Annealing temp. (°C) | Cycle # |
| --- | --- | --- | --- | --- |
| β-Tubulin | GTCGACGGAATCTAGATATAG | CAACATACAGACAGCACGTTG | 50 | 27 |
| Tyrosinase | GGTTCAGCATGTGATGAATG | CAATATGTTAAACCAGTCCAATC | 48 | 30 |
| p14 | CAGTCACTCACACTCGTCTTC | GTGCCACCTTTGCCGCCTTTG | 48 | 30 |
| Fs800 | CATATAGGAATGATCAAATTC | CTATAGTTGGGATTGTTTACC | 48 | 30 |
| FsMucin | GACTGCGGCAATGGTGCAAGTAAA | TCCCAGAGTTCTGGTTGGTTCTGA | 48 | 30 |
| CPEB 2 | CTCTCGATTTGGTAATGTCAG | CAACTATTACAAACAGCATCTTCT | 50 | 36 |
| CPEB 3 | ATGTCAGACGTCGTACGCTCTG | GATTGTTAACACGCAAGCAAG | 50 | 35 |
| *cgh-1* | GTCTGGCTAGATGTGAGACC | GACTCTCAGACATAAATGTGAC | 50 | 36 |
| 6767 | GCAAACTGTTTGGTGGTCTTC | TTTTAAACGTAATCAATCATG | 48 | 27 |
| 15402 | ATGGGGGAAAGCCGAAGCG | ACATCATTTGTGAAACTCATG | 48 | 30 |
| 10395 | ATGACAGTATATTCCTATTTAG | GTGTCTGGGGTATCCATG | 48 | 25 |
| 10435 | TTACGGATATATTTTGGTCTGTC | CAGTTATTACTGTCGAACGAC | 48 | 32 |
| 10401 | CATGTAATACAAACATGGACAG | GTAGTTACTTGATGTGTCACAG | 48 | 32 |
| 1610 | GATTGGAAGTCTCTCTCAAG | AGTAACCCTTAGTTATTCATG | 50 | 28 |
| 8056 | GGCAGTGAATTGAGGTCATC | CCATGGAGGAAGCCTTCAC | 50 | 28 |
| 10548 | CTCAATGGTCTTCACTACTAAC | ATTTACCATCACTAATTCATG | 48 | 30 |
| 21110 | ACAACAGTTTTCTCAGGGCG | CAATATTACAAGATTTACATG | 48 | 30 |
| 10617 | CAACAACATTTCATAAAGGTAG | TACGTACAACAAATATTCATG | 48 | 37 |
| 8987 | GATCACATGTATATCACCAAT | CGACTATGGTTATAGTGTGGC | 48 | 30 |
| 11223 | GAGACTGCTCAGAAAGAAGCA | ATGAACTAGCATATCTATCCG | 48 | 30 |
| 10688 | GATATCTGTAATAACAGTTGTAG | GCTCGAACATCCTCACTTCTC | 48 | 30 |
| 10403 | GTTCCAGAGGCTGTGTCCTGTC | TCACAATCAGCAATGCGATAAG | 48 | 30 |
| 10763 | CATGGACATCATCGGGAAATT | CAGCTGAAGGATTATGGACCT | 48 | 32 |
| 28488 | GCTAACTTCTGACCATGGTATG | TGGTGAAGTAGAAGATACATG | 48 | 35 |
| 21733 | CAGTTGGCATCACTAGTAGGA | TCGGATACTTATTGGAACATG | 48 | 35 |
| 11283 | TACAGTAATATTGGCAATATG | TGAAATGAAAACGTTCACATG | 48 | 30 |
| 33844 | ATGCCAACAGTTGCTGTACC | ATAATTATTATTCTTATCATG | 48 | 35 |
| 10927 | CCGTCCAAGGTCTTCATGATG | TTTTTTCGGTGTATGAACATG | 48 | 30 |
| 11088 | TGCAGGCAAAACTGAAAGTC | CGATACACACTACTGTTTAAAC | 48 | 28 |
| 11779 | CATATTTGGTACCGTGTATATC | CTATACAAATTCAATATCATTAG | 48 | 30 |
| 11055 | GCGATTACAGCCAATTTTGTGG | AACTATGGGCTCACAAACATG | 48 | 35 |
