# Supplementary material for: Transcriptional Analysis of a Unique Set of Genes Involved in Schistosoma mansoni Female Reproductive Biology
Source: PLoS Negl Trop Dis. 2012 Nov 15;6(11):e1907. doi: 10.1371/journal.pntd.0001907 (PMC3499410; doi:10.1371/journal.pntd.0001907)
Supplement: Table S3 — Primers used for quantitative real time PCR. (DOCX) [file pntd.0001907.s004.docx]

Supplementary Table S3: Primers used for quantitative real time PCR. All primers were designed using integrated DNA technologies real time primer design software.

| Gene ID | Forward (5’ – 3’) | Reverse (5’ – 3’) |
| --- | --- | --- |
| β-Tubulin | CACGAGCAGTTAAGCGTTGCAGAA | TATTTGCCGTGACGAGGGTCACAT |
| Tyrosinase | AGTCCATTCTACATGTGGCCGGAA | CTCGAAAGTTTGGCCAACCAGCAT |
| p14 | ACAGTCACTCACACTCGTCTTCTT | GCCATAACCGCTATCACAATC |
| Fs800 | TGGTTATACATATAGGAATGATCAAATTCA | CGTCCACGTGTTTGAAATCG |
| FsMucin | GACTGCGGCAATGGTGCAAGTAAA | ACGCTGTCGACTCAGTTGTTGCTA |
| CPEB 2 | CAGGAAAACTGCCAATTCGAG | CAAACACTGACGTTACCAAATCG |
| *cgh-1* | GCTCATAAGTGGAGTCGAAGAG | TGTAACTGGGTACGTTGCTG |
| 6767 | AGACGGTAAAACAGGTTCCC | GCGCCGGTAATTTTCAAGAAA |
| 15402 | GCGATCTGCTTTACAGTGGTAG | CTAAGTATAGTACGAGCGCCAC |
| 10395 | TCGTAGACATTTTGATCCGGC | TGTACCCAACAGTTTTCCAGG |
| 10435 | TTATTGCCCTGACTGTCCAC | CTCTAGGTGCTGAACTTCTGTG |
| 10401 | TCATCTGCATCATCGTAACCAT | TTCATTCGACTGCACGGTAC |
| 1610 | GATGTTTCCTATTTCTGCTCGG | TTCTCTTAGCACAGCCGC |
| 8056 | GTTTCTTGTCCAACTGTGTATGC | GACCTCAATTCACTGGCATTG |
| 10548 | AAGTTATGGTGAGGCAGACAG | CCACCTAACATAACTCCATGTCG |
| 21110 | TGATATGGCTGAGTTCGGAAC | TCCCTTTTGCATCGTAAGCTC |
| 10617 | AGGAAGGTGGAGAATATAATGATCG | GGCCCTTCAGTCGTTTCTT |
| 8987 | GGAGAAGGTTATGGATTAGTAGGTG | TAGAAGGTATAGGAGTAGGATGAGG |
| 11223 | GTGTCCAAGAGTAGGGAAAGTG | GCCAGTTGCCTTGTTTGATAG |
| 10688 | CTAAAGGCAAATTCCGTTCACG | TTTTCTCTTTTCCACGAGTTTTGA |
| 10403 | CCCTTGTTCGCTCTTTTGTATTC | AGTTTGACAGGACACAGCC |
| 10763 | GATTGTCCATTCACGCCATG | ATCTGCTTCATATCCTTGGGTG |
| 28488 | TTTCCTGCAGAGTGTTATCCG | CCCAAAGACCTTCAACTTGTG |
| 21733 | TGACAGGACGTTCAACTGG | TGAGACAGGATATTGGTGAACG |
| 11283 | GGCAATATGTACATTAGGTGTTGG | GATGCACCAAGAAAAAGTATCGA |
| 33844 | GGCCATGAAAATCTTTATCCTCTTC | AGTCTGTGACAGTTTCAACCTG |
| 10927 | GAATTCAGACAAGTCAGAGGC | CTAACTCCCTCTTTCCACATACG |
| 11088 | TAATAGATCCTGAACCGCC | TGATGAATTGGAGAATCTGT |
| 11779 | ATCATGGATACGATGGTTACAGG | GAAAGGGACCACGAGCATAG |
| 11055 | AGTAGAAACTGAAACCCGGAC | TCGTGCTGTGATCATTCCG |
